# Construction of a GCSCs-Related Prognostic Risk Model Based on Transcriptomic Data

**DOI:** 10.3390/ijms27219669

**Published:** 2026-10-28

**Authors:** Xin Hu, Shiqi Fan, Naiqiang Liu, Muchen Fan, Yewen Wu, Guang Shi, Yangguang Su, Lei Liu, Xiujie Chen

**Affiliations:** College of Bioinformatics Science and Technology, Harbin Medical University, Harbin 150081, China; 2025020449@hrbmu.edu.cn (X.H.); 2025020484@hrbmu.edu.cn (S.F.); liunaiqiang@hrbmu.edu.cn (N.L.); 2024020592@hrbmu.edu.cn (M.F.); 2019158016@hrbmu.edu.cn (Y.W.); 2025020438@hrbmu.edu.cn (G.S.); 202401206@hrbmu.edu.cn (Y.S.)

**Keywords:** gastric cancer, gastric cancer stem cells, single-cell RNA sequencing, prognostic risk model, tumor immune microenvironment, immunotherapy

## Abstract

Gastric Cancer Stem Cells (GCSCs) contribute to tumor progression and treatment resistance, but their heterogeneity and tumor microenvironment interactions remain unclear. We analyzed GSE163558 single-cell RNA-sequencing data from 10 gastric cancer samples and integrated GCSC-related genes with The Cancer Genome Atlas Stomach Adenocarcinoma (TCGA-STAD) training cohort (*n* = 448) and two independent external validation cohorts, GSE62254 (*n* = 300) and GSE84437 (*n* = 483), to construct and validate a six-gene prognostic risk model. Cell–cell communication, metabolic activity, immune infiltration, immunotherapy response, and drug sensitivity were evaluated. Nine cell populations, including a putative GCSC population, were identified. GCSCs showed higher inferred oxidative phosphorylation activity and extensive predicted interactions with tumor-associated cells. The model stratified patients into distinct prognostic groups. High-risk tumors exhibited enrichment of progression-related pathways, including epithelial–mesenchymal transition, and distinct immune features. In PRJEB25780 (*n* = 78), higher risk scores were associated with immunotherapy non-response, whereas an exploratory analysis of GSE91061 (*n* = 49) showed a similar trend. The model may support prognostic stratification and provide insights into potential associations with therapeutic response, pending prospective validation.

## 1. Introduction

Gastric cancer (GC) is one of the most important malignancies of the digestive system worldwide. It is estimated that approximately 969,000 new cases of GC and 660,000 GC-related deaths occur worldwide each year, ranking GC fifth in both cancer incidence and cancer-related mortality, with a particularly high disease burden in East Asia [1]. Although advances in surgery, chemotherapy, targeted therapy, and immunotherapy have improved patient outcomes to some extent, the overall prognosis of patients with advanced GC remains unsatisfactory [2,3]. High tumor heterogeneity, late-stage diagnosis, recurrence and metastasis, treatment resistance, and the lack of reliable prognostic and therapeutic response biomarkers continue to hinder precision treatment for GC.

Cancer stem cells (CSCs) are a subpopulation of tumor cells characterized by self-renewal, multilineage differentiation, and tumor repopulating capacities and are closely associated with tumor heterogeneity, recurrence, metastasis, and treatment resistance [4,5]. GCSCs may also contribute to the formation and remodeling of the tumor microenvironment (TME) through metabolic reprogramming and interactions with immune and stromal cells [6,7]. However, GCSCs do not constitute a uniform and stable cell population, and their molecular characteristics and functional states may vary substantially across cells, samples, and disease stages. Conventional bulk transcriptomic analyses mainly reflect the average expression profiles of mixed cell populations and are therefore limited in their ability to accurately identify GCSC states and their interactions with other cell populations. scRNA-seq provides a powerful approach for dissecting the heterogeneity and microenvironmental regulatory networks of GCSCs [8,9]. Nevertheless, systematic studies integrating the single-cell features of GCSCs with the prognosis of patients with GC remain limited.

Nevertheless, the metabolic characteristics and cell–cell communication patterns of transcriptionally defined GCSC populations remain insufficiently characterized, and their potential relationships with patient-level prognosis have not been systematically established. This represents an important gap between the single-cell biological characterization of GCSCs and their potential clinical relevance.

At present, prognostic assessment for patients with GC mainly relies on the TNM staging system, Lauren classification, and other clinicopathological parameters. The Cancer Genome Atlas further classified GC into four molecular subtypes: Epstein–Barr viruspositive, microsatellite instability, genomically stable, and chromosomal instability, thereby advancing the understanding of the molecular heterogeneity of GC [10]. However, these classification systems remain insufficient to fully capture interpatient differences in cellular composition, functional states, and therapeutic response [11]. In recent years, prognostic models based on gene expression signatures have increasingly been used for risk stratification in GC [12]. However, most existing models rely on bulk transcriptomic data alone, lack a clear single-cell biological basis, and rarely consider the potential contribution of GCSCs to model development. Furthermore, the extent to which GCSC-related molecular signatures are associated with the immune microenvironment and clinical treatment response remains unclear. In addition, immune checkpoint inhibitors have become an important treatment option for a subset of patients with advanced GC, although only a proportion of patients achieve durable clinical benefit. Therefore, the development of a biologically informed prognostic risk model that integrates GCSC-related molecular features with patient survival and immune characteristics may have important clinical relevance [13].

In this study, we integrated the GSE163558 scRNA-seq dataset, which was generated from human gastric cancer specimens and provides single-cell transcriptomic profiles of multiple tumor samples, together with transcriptomic and clinical data from the TCGA-STAD training cohort and two independent prognostic validation cohorts. This dataset was selected because it enables high-resolution characterization of gastric cancer cellular heterogeneity and provides an appropriate resource for identifying putative GCSC populations and their associated molecular features. First, a putative GCSC population was identified at single-cell resolution, followed by analyses of its cell–cell communication network and metabolic characteristics. GCSC-related genes were then identified, and a six-gene prognostic risk model was constructed using univariable Cox regression, LASSO-penalized Cox regression, and multivariable Cox regression. Its prognostic stratification performance and independent prognostic value were evaluated in the training and external validation cohorts. Subsequently, functional pathways and immune microenvironmental features were compared between the high- and low-risk groups [14], and the association between the risk score and observed immunotherapy response was assessed in the PRJEB25780 gastric cancer immunotherapy cohort and the GSE91061 melanoma anti-PD-1 cohort [15,16]. Conventional chemotherapeutic agents, including 5-fluorouracil, oxaliplatin, irinotecan, and paclitaxel, remain important treatment options for gastric cancer. However, GCSCs have been implicated in resistance to conventional chemotherapy, highlighting the need to investigate their potential association with therapeutic sensitivity [17]. Finally, in silico drug sensitivity analysis was performed to explore associations between the risk score and predicted drug sensitivity, with particular attention to these clinically relevant chemotherapeutic agents. By linking the single-cell characteristics of GCSCs to patient-level prognostic and immune features, this study aimed to address the gap between GCSC biological heterogeneity and its potential clinical significance, while providing a foundation for further investigation of GCSC-related prognostic biomarkers in GC.

## 2. Results

### 2.1. Identification and Gene Distribution of GCSCs

**Figure 1:**
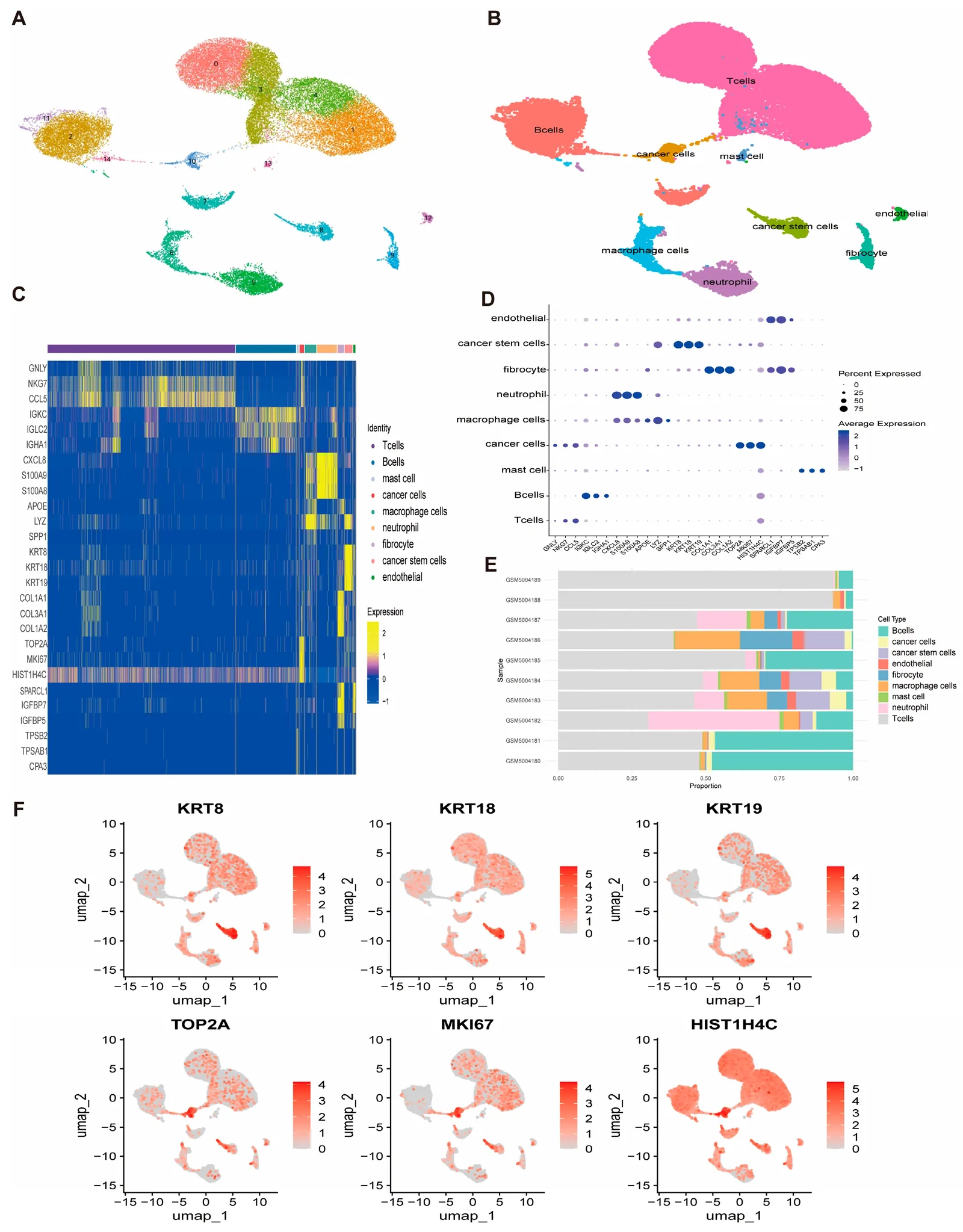
Single-cell landscape of gastric cancer and identification of GCSCs: (**A**) UMAP plot showing the unsupervised clustering of cells based on their transcriptomic profiles. (**B**) UMAP plot showing the annotation of nine major cell populations based on established cell-type marker genes. Cluster numbers and corresponding cell types are labeled in the figure. (**C**) A heatmap illustrates the top three marker genes for nine cell types. (**D**) A bubble plot displays the marker genes of the nine cell types. (**E**) Stacked bar plot illustrating the distribution of the nine cell types across scRNA-seq samples. (**F**) Expression patterns of representative GCSC-associated and epithelial cancer cell markers across the annotated cell populations.

To accurately identify GCSCs and characterize their gene expression distribution, the GSE163558 dataset from the Gene Expression Omnibus (GEO) database was used to delineate cellular subpopulations. This dataset comprises multiple cell types within the gastric cancer microenvironment, including the GCSC population of primary interest. To minimize the potential impact of technical noise on downstream analyses, stringent preprocessing and quality control procedures were applied to remove low quality cells and genes with aberrant expression profiles. Unsupervised clustering subsequently identified 15 cell clusters (Figure 1A), which were further annotated into nine distinct cell subpopulations (Figure 1B). Cell subpopulations were identified based on the expression of established cell-type-specific marker genes. GCSCs were characterized by the expression of CD44, CD24, KRT18, KRT8, and ALDH1A1. T cells were identified using PTPRC, CD3D, CD4, CD3E, and CD8A, whereas B cells were defined by CD19, CD79A, MS4A1, TAGLN2, and CD5. Fibroblasts were annotated based on ACTA2, PDGFRA, PDGFRB, COL1A1, and COL3A1. Endothelial cells were identified using SPARCL1, IGFBP7, IGFBP5, MRC1, and MSR1. Neutrophils were characterized by S100A9, S100A8, CXCL8, FCGR3B, and CXCR2. Gastric cancer cells were defined by TOP2A, MKI67, HIST1H4C, EPCAM, and FIGR. Mast cells were identified using TPSB2, CPA3, and TPSAB1, whereas macrophages were characterized by C1QA, LYZ, APOE, CD68, and SPP1.

Nine major cell types were identified in gastric cancer. The expression patterns of representative marker genes across these cell types are presented in the heatmap (Figure 1C) and bubble plot (Figure 1D), whereas their relative proportions across individual samples are shown in Figure 1E. Substantial interpatient heterogeneity was observed in the abundance of GCSCs. In some patients, GCSCs accounted for more than 10% of the total tumor cell population, whereas in others, their proportion was below 2%. This variation may be associated with differences in tumor progression, disease stage, and previous treatment history.

Comparison of marker gene expression between GCSCs and conventional cancer cells revealed distinct transcriptional patterns (Figure 1F). Genes such as KRT8 and KRT18 were preferentially expressed in GCSCs but showed relatively low expression in cancer cells. Conversely, proliferation-associated genes, including TOP2A and MKI67, were highly expressed in cancer cells but exhibited lower expression in GCSCs. These differences suggest distinct biological states between the two cell populations: GCSCs may be more closely associated with the maintenance of stem-like properties and self-renewal capacity, whereas conventional cancer cells display stronger proliferative activity. Further investigation of these transcriptional differences may improve our understanding of gastric cancer development and provide important clues for the development of therapeutic strategies specifically targeting GCSCs.

### 2.2. Cell–Cell Communication Analysis

TME is characterized by extensive interactions among multiple cell populations, and alterations in intercellular communication may compromise the efficacy of anticancer therapies. In this study, the CellChat algorithm was used to comprehensively characterize signaling interactions within the TME, with particular emphasis on the communication between GCSCs and the other eight identified cell types. Elucidating these intercellular signaling networks is important for understanding tumor biology and informing therapeutic strategies.

**Figure 2:**
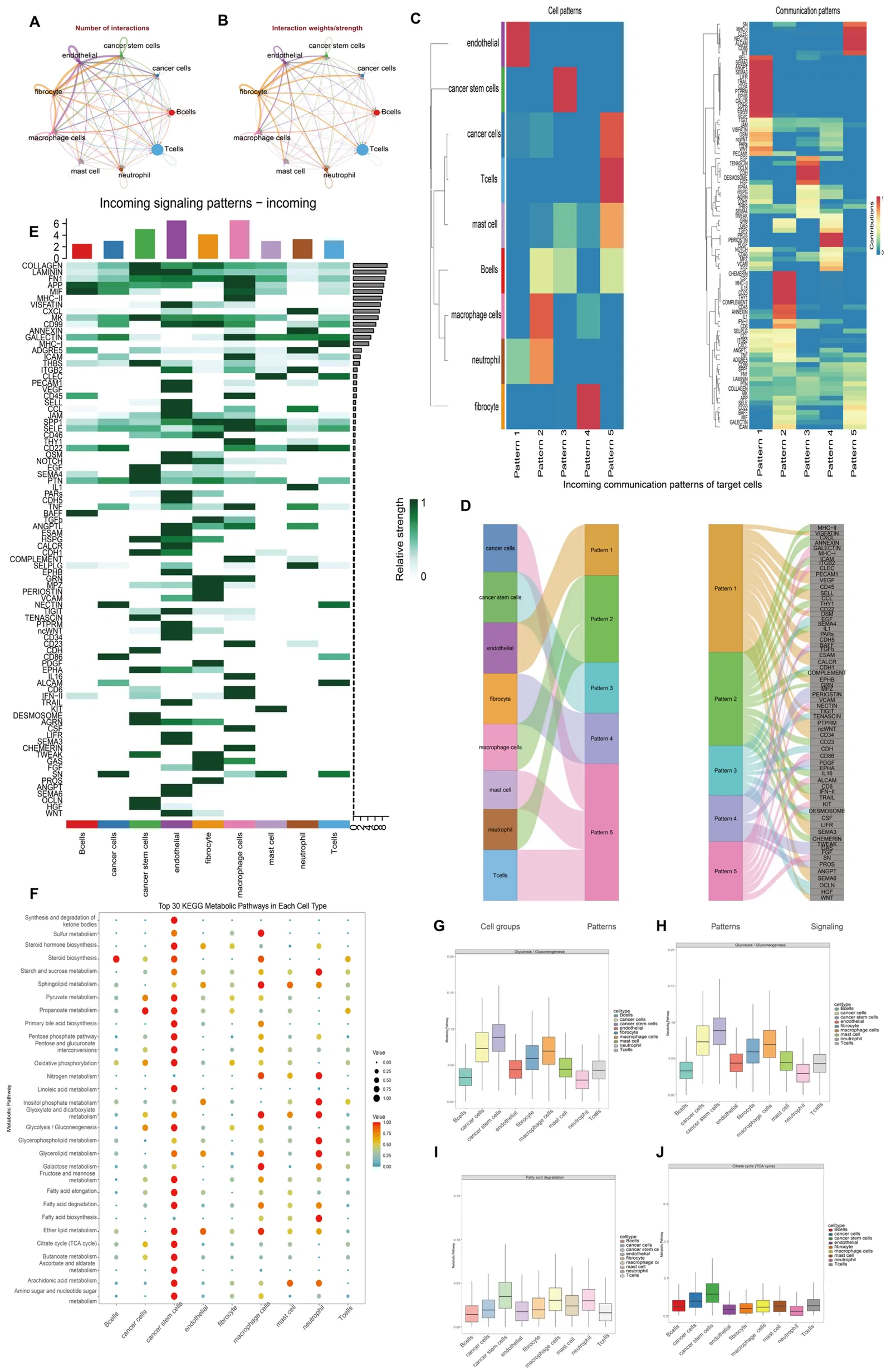
Cell–cell communication and metabolic functional analysis of GCSCs: (**A**,**B**) Circular plot showing interaction counts among the nine cell types. (**C**) Heatmaps showing incoming signaling patterns among the nine cell types. (**D**) Sankey diagrams illustrating the incoming signaling patterns among the nine cell types. (**E**) Clustering heatmaps showing incoming signals across cell types. (**F**) Scatter plot of the top 30 KEGG metabolic pathways ranked by enrichment scores across nine cell types. (**G**–**J**) Box plots showing metabolic activity scores for glycolysis, oxidative phosphorylation, fatty acid degradation, and TCA cycle.

By quantifying both the number and strength of inferred cell–cell interactions, we found that GCSCs exhibited extensive communication with cancer cells and immune cell populations, particularly macrophages, neutrophils, B cells, and T cells (Figure 2A,B). These findings suggest that GCSCs may actively participate in intercellular signaling and molecular exchange within the TME, thereby contributing to its establishment, maintenance, and dynamic remodeling.

Subsequently, to further characterize the coordinated signaling functions of GCSCs across multiple cell populations and pathways, communication patterns were identified using clustering analyses evaluated by the cophenetic coefficient and silhouette score.

Five distinct patterns were selected for both incoming and outgoing signaling. In the incoming signaling analysis, GCSCs were predominantly associated with pattern 3 and received signals mainly through the EGF, TENASCIN, OCLN, CDH, DESMOSOME, and HGF pathways, suggesting that these pathways may contribute to the regulation of GCSC biological behavior (Figure 2C). In the outgoing signaling analysis, GCSCs were primarily assigned to pattern 4 and transmitted signals through pathways including SEMA3, OCLN, KIT, CDH1, CDH, MK, and DESMOSOME, thereby facilitating communication and coordination with other cell populations (Figure S1A).

The Sankey diagrams of cell–cell communication (Figure 2D and Figure S1B) visualize the incoming and outgoing signaling patterns. The clustered heatmaps further illustrate the incoming and outgoing communication patterns among the different cell types, as well as the signaling interactions between them. In the incoming signaling pattern, GCSCs received signals from other cell populations through multiple pathways, including COLLAGEN, LAMININ, MK, THBS, EGF, SEMA4, PTN, HSPG, CDH1, TENASCIN, CDH, EPHA, DESMOSOME, AGRN, SEMA6, OCLN, and HGF (Figure 2E). In contrast, in the outgoing signaling pattern, GCSCs transmitted signals to other cell types through pathways including MIF, MK, MHCI, CD46, PARs, CDH1, CDH, PDGF, KIT, DESMOSOME, AGRN, SEMA3, and OCLN (Figure S1C).

### 2.3. Metabolic Function Analysis of Cells

TME is a dynamic network composed of tumor cells, immune cells, stromal cells, and other cellular components. Through their involvement in multidimensional metabolic processes, including energy production, biosynthesis, and molecular transformation, these cells profoundly influence tumor growth, invasion, metastasis, and therapeutic sensitivity. In this study, metabolic pathway enrichment analysis was performed using the scRNA-seq data. Metabolic enrichment scores were calculated for each cell population, and the 30 most highly enriched KEGG metabolic pathways were identified and visualized in a dot plot (Figure 2F). The plot illustrates the distribution of metabolic pathway activity scores across nine cell types, including B cells, cancer cells, GCSCs, endothelial cells, fibroblasts, macrophages, mast cells, neutrophils, and T cells.

Notably, GCSCs exhibited higher enrichment scores than the other cell types across approximately 20 active metabolic pathways, including oxidative phosphorylation, glycolysis, fatty acid degradation, the tricarboxylic acid cycle, and amino sugar and nucleotide sugar metabolism. The relative enrichment of glycolysis, oxidative phosphorylation, the TCA cycle, and fatty acid degradation across the different cell types was further illustrated using box plots.

Compared with conventional tumor cells and immune cells, GCSCs exhibited higher glycolytic activity (Figure 2G). This finding suggests that GCSCs may enhance glycolysis to generate sufficient energy in the form of ATP and biosynthetic intermediates to support their cellular activities. Analysis of oxidative phosphorylation further showed that GCSCs had substantially higher pathway activity than the other cell types (Figure 2H), suggesting a strong capacity to maintain mitochondrial function and a stable energy supply.

GCSCs also exhibited the highest activity in fatty acid degradation (Figure 2I) and the tricarboxylic acid cycle (Figure 2J) among all analyzed cell types. These findings indicate that GCSCs may rely extensively on lipid metabolism and the TCA cycle. Such metabolic characteristics may support GCSC survival, resistance to cell death, and the development of therapeutic resistance.

GCSCs exhibit a distinctive metabolic profile characterized by the concurrent activation of glycolysis, oxidative phosphorylation, and fatty acid metabolism. This metabolic pattern is consistent with the metabolic plasticity previously reported in cancer stem cell populations, which can flexibly utilize both glycolytic and mitochondrial oxidative pathways to maintain energy production under heterogeneous tumor conditions [18]. Enhanced glycolysis may promote rapid glucose utilization and lactate production, thereby contributing to acidification of the tumor microenvironment. Such an acidic microenvironment may facilitate tumor cell invasion and dissemination into surrounding tissues. In particular, the increased oxidative phosphorylation activity observed in GCSCs suggests that these cells may maintain strong mitochondrial metabolic capacity. Previous studies have demonstrated that certain CSC populations exhibit preferential dependence on oxidative phosphorylation rather than glycolysis, highlighting mitochondrial metabolism as a potential therapeutic vulnerability [19]. Through oxidative phosphorylation and fatty acid degradation, GCSCs may not only maintain a stable energy supply but also generate biosynthetic intermediates required for the production of nucleotides, amino acids, and other essential cellular components. These metabolic processes may be critical for sustaining self-renewal, preserving stem-like properties, and promoting resistance to anticancer therapies.

This metabolic flexibility may enable GCSCs to survive under nutrient limited conditions within tumors. In addition, metabolites released by GCSCs, including lactate and ATP, may modulate the functions of surrounding immune cells. These metabolites may impair T cell-mediated antitumor responses, promote macrophage recruitment, and contribute to the establishment of an immunosuppressive microenvironment that supports tumor growth and progression.

Collectively, these findings provide a metabolic perspective on the survival and functional adaptation of GCSCs within the TME and offer potential mechanistic insights into the therapeutic resistance of GCSCs.

### 2.4. Construction of a GCSC-Related Gene Prognostic Risk Model

**Figure 3:**
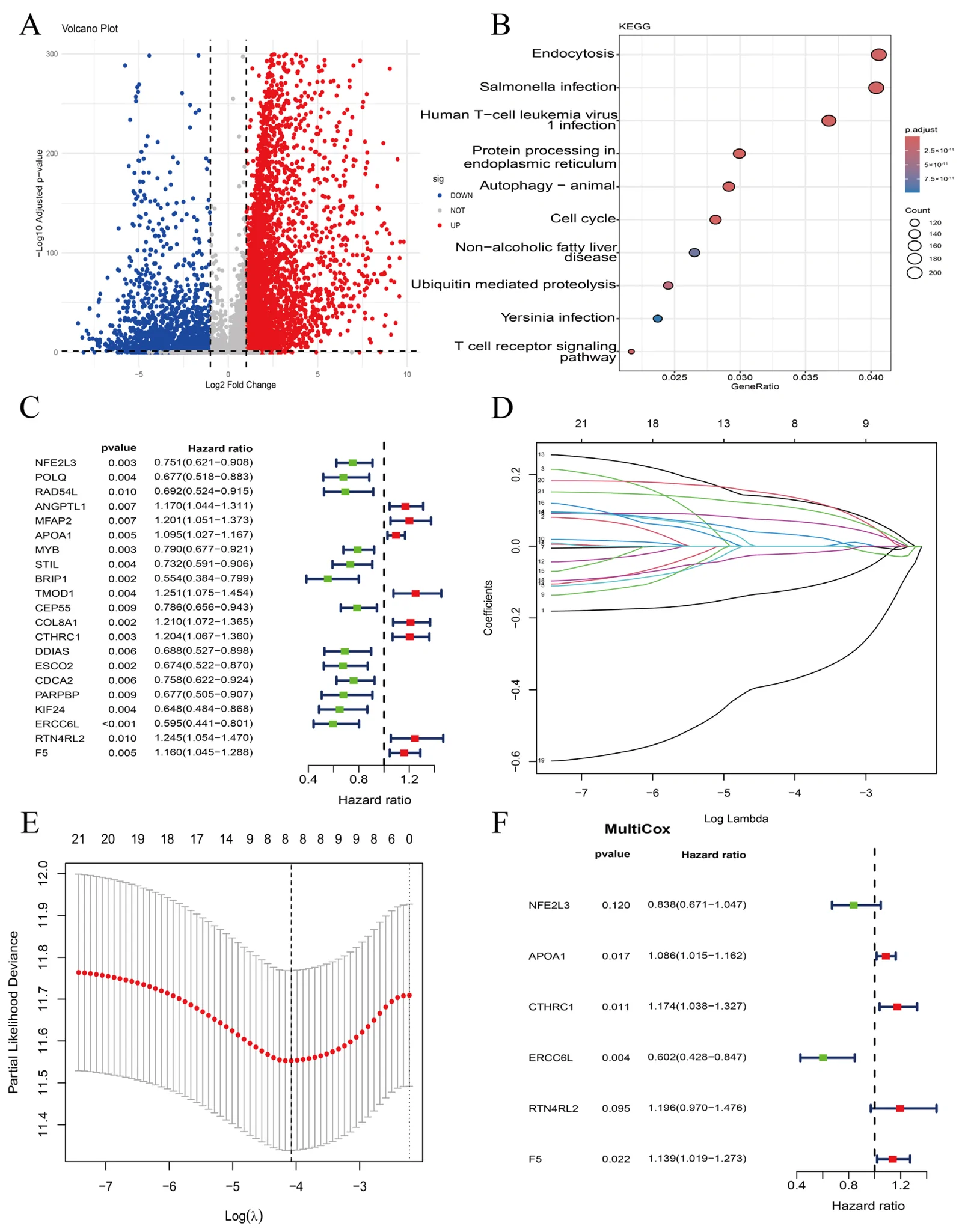
Construction of a GCSC-related Gene Prognostic Risk Model: (**A**) Volcano plot of GCSC-related genes, highlighting differentially expressed genes. (**B**) Bubble plot of KEGG enrichment analysis for GCSC-specific genes. (**C**) Forest plot showing 21 prognostic genes screened by univariate Cox regression analysis. (**D**) Selection of the optimal lambda via cross-validation in LASSO regression. (**E**) Trajectory of variable coefficients in LASSO regression. (**F**) Forest plot showing 6 prognostic genes screened by multivariate Cox regression analysis.

To investigate the clinical prognostic value of GCSC-associated genes, differential expression analysis was first performed using the scRNA-seq data. A total of 883 GCSCs and 513 non-GCSC tumor epithelial cells were included in the differential expression analysis. A total of 10,869 genes that were significantly upregulated in the GCSC subpopulation were identified using the thresholds of |log2FC| > 1 and FDR < 0.05. The differential expression results were visualized using a volcano plot (Figure 3A), which clearly demonstrated the expression changes in GCSC-associated genes and provided a basis for subsequent analyses. KEGG pathway enrichment analysis was then conducted to functionally annotate these differentially expressed genes and identify the biological pathways in which they were significantly enriched (Figure 3B).

The results showed that GCSC-associated genes were significantly enriched in several important pathways, including endocytosis, Salmonella infection, human T cell leukemia virus 1 infection, protein processing in the endoplasmic reticulum, Yersinia infection, and autophagy in animals. Cell cycle-related enrichment was also observed in Figure 3B. This finding does not necessarily contradict the relatively lower proliferative characteristics of GCSCs, as pathway enrichment reflects the involvement of cell cycle-related genes rather than directly indicating increased proliferative activity. These findings suggest that GCSCs may be involved in multiple biological processes, including endocytosis, host–pathogen interactions, immune responses, cellular metabolism, and inflammatory responses.

The enrichment results further revealed the distinctive molecular characteristics of GCSCs across several key biological processes and provided pathway-level insights into the mechanisms underlying stemness maintenance, therapeutic resistance, and immune evasion. Together with the subsequent construction and validation of a GCSC-related prognostic risk model, these findings established a functional genomic basis for identifying molecular biomarkers for survival prediction in patients with gastric cancer.

After identifying the GCSC-associated differentially expressed genes and their enriched functional pathways, we further investigated their clinical prognostic value. Genes overlapping between the GCSC-associated differentially expressed genes and the differentially expressed genes identified in the TCGA gastric cancer cohort were selected, yielding a gene set that was both closely associated with GCSCs and differentially expressed in TCGA-STAD. After integrating gene expression data with clinical information, univariate Cox regression analysis was performed using *p* < 0.01 as the significance threshold to evaluate the association between each gene and overall survival. This analysis identified 21 prognostic genes significantly associated with patient survival. These genes are presented in Figure 3C, together with their *p* values, hazard ratios, and 95% confidence intervals. The complete univariate Cox regression results for all 21 prognostic genes, including gene annotations, regression coefficients, hazard ratios, 95% confidence intervals, and *p* values, are provided in Supplementary Table S1.

To improve model specificity and prognostic accuracy, LASSO-Cox regression analysis was subsequently performed. Based on the changes in regression coefficients across log-transformed λ values (Figure 3D) and selection of the optimal λ value (Figure 3E), eight candidate prognostic genes were retained. Multivariate Cox regression analysis was then conducted to account for the combined effects of multiple genes and other potential prognostic factors. Using the same significance threshold of *p* < 0.01, six core prognostic genes were ultimately identified: NFE2L3, APOA1, CTHRC1, ERCC6L, RTN4RL2, and F5 (Figure 3F).

These six genes were used to construct a GCSC-related prognostic risk model. The model calculates an individual risk score for each patient based on the expression levels of the six genes, thereby enabling stratification of patients according to their predicted clinical outcomes. This risk model may assist in prognostic assessment and provide a basis for individualized treatment planning in patients with gastric cancer. Patients classified into different risk groups may require distinct therapeutic strategies, although the clinical utility of the model should be further validated in prospective cohorts.

The regression coefficients of the genes included in the risk model are presented in Table 1. The risk score was calculated based on the expression levels and corresponding regression coefficients of the six GCSC-associated genes. Using the median risk score as the cutoff, patients in the TCGA-STAD training cohort were stratified into high- and low-risk groups.

**Figure 4:**
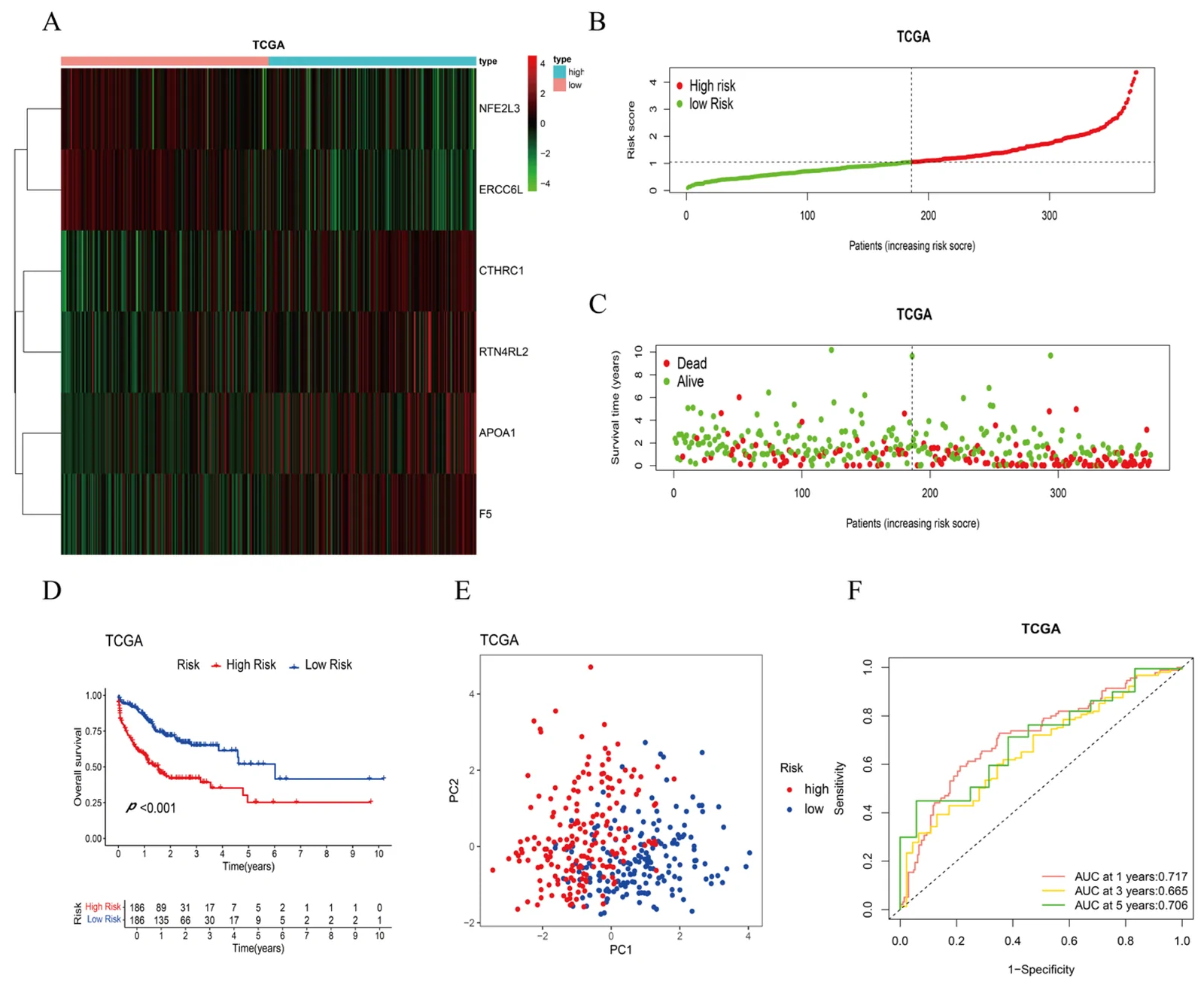
Construction of a GCSC-related Gene Prognostic Risk Model: (**A**) Heatmap of risk scores in the training cohort, where the color gradient represents gene expression intensity. (**B**) Risk score distribution plot for the training cohort, ordered by increasing risk score. (**C**) Risk scatter plot correlating risk scores with survival outcomes in the training cohort. (**D**) KM survival curves demonstrate significantly shorter OS in the high-risk group compared to the low-risk group (*p* < 0.001). (**E**) PCA scatter plot highlights the differentiation capacity of the model, with high- and low-risk groups showing distinct clustering in the principal component space. (**F**) Time-dependent ROC curves validate the model’s predictive performance for 1-, 3-, and 5-year survival in the TCGA training cohort.

The heatmap (Figure 4A) illustrates the expression patterns of the six model genes and the distribution of patient risk groups. Importantly, the six genes included in the model did not exhibit identical expression trends across risk groups because they contributed differently to the risk score according to their regression coefficients. Among these genes, CTHRC1, RTN4RL2, APOA1, and F5 showed positive coefficients and therefore contributed to increased risk scores when their expression levels increased. In contrast, NFE2L3 and ERCC6L showed negative coefficients, indicating that higher expression of these genes was associated with reduced risk scores within the model. Therefore, the heatmap reflects the combined contribution of both risk-promoting and protective genes rather than a uniform expression pattern of all six genes. Cluster analysis revealed distinct gene expression profiles between the high- and low-risk groups. The risk score distribution plot (Figure 4B) shows that patients classified into the high-risk group generally had higher risk scores. The survival status scatter plot (Figure 4C) further demonstrates the relationship between risk score and survival outcome, with patients exhibiting higher risk scores tending to have shorter survival times. Collectively, these findings provide preliminary evidence that the six-gene risk score may have prognostic value in patients with gastric cancer.

Analysis of the training cohort showed that patients in the high-risk group had a substantially shorter survival time than those in the low-risk group, with estimated survival times of approximately 1 year and 6 years, respectively (Figure 4D). The difference between the two groups was statistically significant (*p* < 0.001).

To investigate the biological differences between the high- and low-risk groups, principal component analysis (PCA) was performed to reduce the dimensionality of the gene expression data. The samples from the two risk groups exhibited a clear separation in the PCA plot (Figure 4E), indicating that the risk model captured distinct gene expression patterns between patients with different risk levels.

Time-dependent receiver operating characteristic (ROC) curve analysis was further conducted to evaluate the prognostic performance of the model (Figure 4F). The areas under the ROC curves for predicting 1, 3, and 5 year overall survival were 0.717, 0.665, and 0.706, respectively. These results indicate that the model demonstrated moderate prognostic discrimination at different time points and may provide useful information for patient risk stratification and individualized clinical management.

### 2.5. Validation of the GCSC-Related Gene Prognostic Risk Model

To assess the reliability and generalizability of the GCSC-associated prognostic risk model, two independent gastric cancer cohorts, GSE62254 and GSE84437, were selected for external validation after data preprocessing. For each validation cohort, patient risk scores were calculated using the same scoring formula and regression coefficients applied in the training cohort. Patients were then stratified into high- and low-risk groups according to their risk scores.

Applying an identical scoring strategy across the training and validation cohorts ensured methodological consistency and reduced the potential influence of cohort specific characteristics. This external validation framework enabled an objective evaluation of the stability, reproducibility, and potential clinical applicability of the prognostic model in independent patient populations.

**Figure 5:**
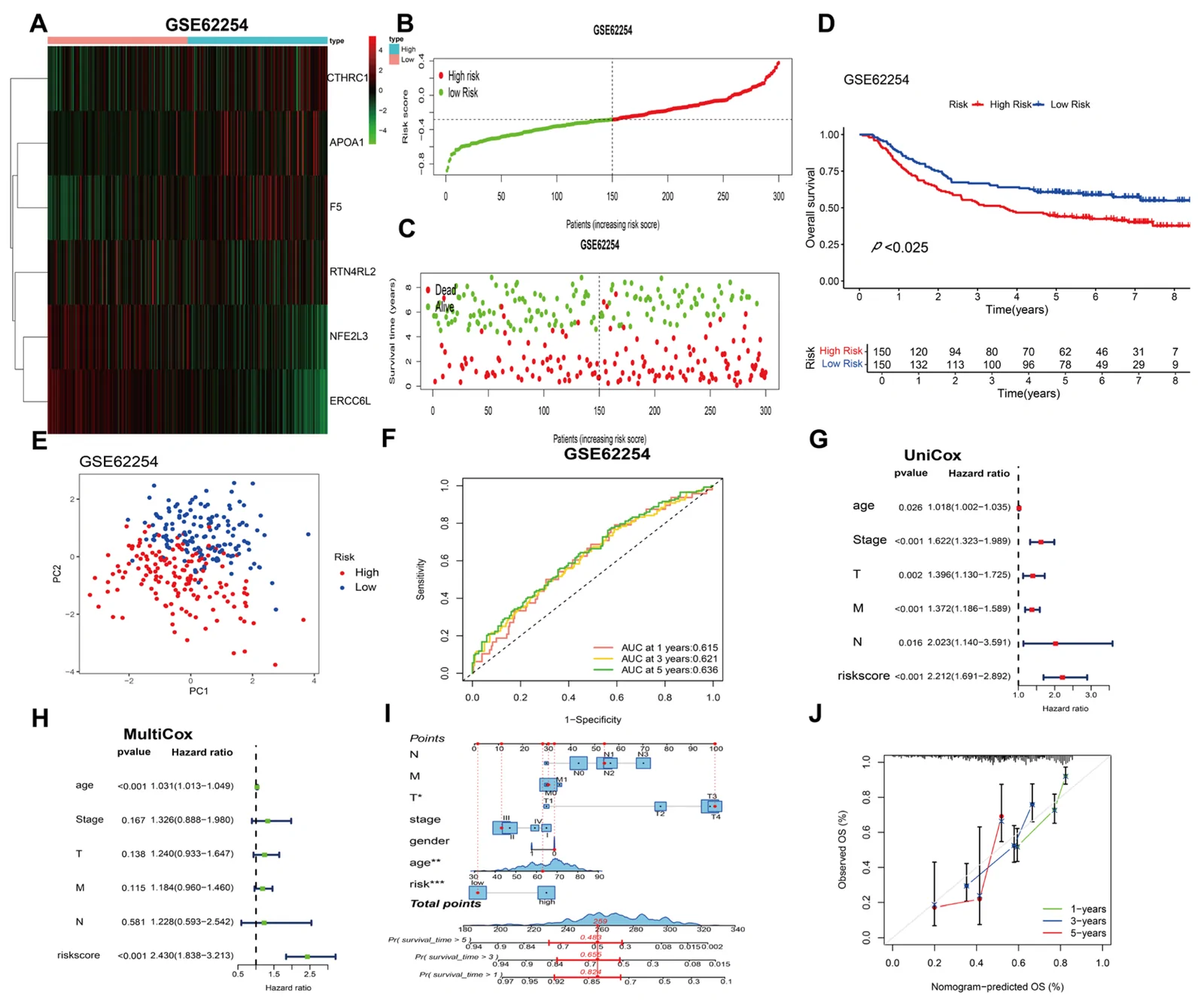
Validation of the GCSC-related Gene Prognostic Risk Model: (**A**) Risk heatmap of GSE62254, where the top color bar distinguishes high- and low-risk patients. (**B**) Risk score distribution plot for GSE62254, with patients ordered by ascending risk scores. (**C**) Risk scatter plot correlating risk scores with survival outcomes in the GSE62254 cohort. (**D**) KM survival curves for the GSE62254 cohort. (**E**) PCA plots of high- and low-risk samples in the GSE62254 cohort. (**F**) Time-dependent ROC curves for 1-, 3-, and 5-year survival prediction in the GSE62254 cohort. (**G**) Forest plot of univariate Cox regression analysis incorporating traditional clinical factors. (**H**) Forest plot of multivariate Cox regression analysis integrating clinical factors. (**I**) Nomogram for predicting survival probabilities. Blue boxes indicate predictor categories, and blue density curves show the distributions of age and total points. Red dotted lines and annotations illustrate the calculation of risk scores and predicted survival probabilities for a randomly selected patient. *, **, and *** indicate statistical significance at *p* < 0.05, *p* < 0.01, and *p* < 0.001, respectively. (**J**) Calibration curve validating the predictive accuracy of the nomogram.

In the GSE62254 validation cohort, the risk heatmap (Figure 5A) illustrates the expression patterns of the six key genes, including APOA1, F5, and CTHRC1. Comparison between the high- and low-risk groups revealed distinct expression patterns among these genes. For example, CTHRC1 was highly expressed in the high-risk group but showed relatively low expression in the low-risk group, suggesting that its expression may be associated with patient risk stratification.

The risk score distribution plot (Figure 5B) shows the relationship between patient ranking, risk scores, and risk group classification. Risk scores increased progressively among patients in the high-risk group, whereas relatively lower scores were observed in the low-risk group, indicating that the model effectively stratified patients into different risk categories. The survival status scatter plot (Figure 5C) further demonstrated the relationship between risk score and survival outcome. Death events were more frequently observed among patients with higher risk scores, whereas surviving patients were more concentrated in the lower risk region. These findings suggest that higher risk scores were associated with shorter survival in the GSE62254 validation cohort.

Analysis of the GSE84437 validation cohort yielded results broadly consistent with those obtained in GSE62254. The risk heatmap (Figure S2A) showed a clear separation in gene expression patterns between the high- and low-risk groups. Key genes such as APOA1 and CTHRC1 displayed expression differences between the two groups that were consistent with those observed in the training cohort and the GSE62254 validation cohort, supporting the reproducibility of the risk model. In the risk score distribution plot (Figure S2B), risk scores increased progressively as patients were ordered from low- to high-risk, with patients in the high-risk group exhibiting markedly higher scores than those in the low-risk group. These findings indicate that the model maintained its ability to stratify patients across independent datasets. The survival status scatter plot (Figure S2C) further showed that death events were more frequent among patients with higher risk scores, whereas surviving patients were more concentrated in the lower risk region. This pattern was consistent with the findings from GSE62254 and indicated that higher risk scores were associated with poorer survival outcomes.

Survival analyses provided further evidence for the prognostic value of the risk score. In the GSE62254 validation cohort, KM survival analysis (Figure 5D) demonstrated a significant difference in overall survival between the high- and low-risk groups. The estimated median survival was approximately 6 years in the high-risk group and 11 years in the low-risk group (*p* < 0.025). These results suggest that the six genes GCSC-associated risk model can reproducibly distinguish patients with different survival outcomes and may provide useful information for prognostic stratification and individualized clinical management.

PCA was performed to further evaluate the ability of the risk score to distinguish patients with different risk levels (Figure 5E). The gene expression profiles of patients in the high- and low-risk groups formed two relatively distinct clusters in the PCA plot. This separation supports the discriminatory capacity of the risk model and suggests that the model captures gene expression patterns associated with patient prognosis.

Time-dependent ROC curve analysis was also conducted to assess the prognostic performance of the risk score at different time points (Figure 5F). The AUC values for predicting 1-, 3-, and 5-year overall survival were 0.615, 0.621, and 0.636 respectively. The gradual increase in AUC over time suggests that the model showed slightly better discrimination for longer term survival, although its overall predictive performance in this cohort was modest.

Analysis of the GSE84437 cohort similarly supported the prognostic relevance of the risk score. KM survival analysis showed that patients in the high-risk group had significantly poorer overall survival than those in the low-risk group (*p* < 0.001; Figure S2D), indicating that the risk score could distinguish patients with different survival outcomes. PCA was then used to evaluate the separation of the two risk groups (Figure S2E). In the principal-component space, high- and low-risk patients were distributed in relatively distinct regions, suggesting that the model captured different gene expression characteristics between the two groups.

Time-dependent ROC curve analysis in the GSE84437 cohort showed AUC values of 0.582, 0.599, and 0.611 for predicting 1-, 3-, and 5-year overall survival, respectively (Figure S2F). These results indicate that the prognostic discrimination of the risk score increased slightly over time, with modest predictive performance for both short- and long-term survival.

Subsequently, to further evaluate the independent prognostic value of the risk score, univariate and multivariate Cox regression analyses were performed by integrating the risk score with clinical variables, including age, overall tumor stage, T stage, M stage, and N stage. Univariate Cox regression analysis (Figure 5G) showed that the risk score was significantly associated with patient prognosis (HR = 2.212, 95% CI: 1.691–2.892, *p* < 0.001). Its hazard ratio was higher than those of several conventional clinical variables, including age (HR = 1.018, 95% CI: 1.002–1.035, *p* = 0.026) and overall tumor stage (HR = 1.622, 95% CI: 1.323–1.989, *p* < 0.001), providing preliminary evidence that the risk score was closely associated with survival outcomes.

In the multivariate Cox regression analysis (Figure 5H), the risk score remained a significant independent prognostic factor after adjustment for other clinical variables (HR = 2.430, 95% CI: 1.838–3.213, *p* < 0.001). Its hazard ratio was higher than those of several conventional clinical indicators, including age (HR = 1.031, 95% CI: 1.013–1.049, *p* < 0.001) and overall tumor stage (HR = 1.326, 95% CI: 0.888–1.980, *p* = 0.167). These findings provide strong evidence that the risk score can independently predict patient survival beyond established clinicopathological factors, suggesting that it may serve as an independent prognostic biomarker and provide additional information for clinical risk assessment.

Based on these findings, a prognostic nomogram was constructed (Figure 5I). The nomogram integrated the risk score with multiple clinical variables, including patient age and tumor stage, and assigned a corresponding point value to each factor to estimate individual survival probabilities. Calibration curves were subsequently generated to evaluate the predictive accuracy of the nomogram (Figure 5J). The predicted 1-, 3-, and 5-year survival probabilities were generally consistent with the observed outcomes, indicating good agreement between the nomogram predictions and actual patient survival.

In summary, the GCSC-associated prognostic risk model demonstrated reproducible predictive performance across multiple independent validation cohorts and retained independent prognostic significance after adjustment for conventional clinical variables. The model effectively stratified patients with gastric cancer into high- and low-risk groups and may provide a useful framework for individualized prognostic assessment and clinical decision making. However, further prospective and multicenter validation is required before its routine clinical application.

### 2.6. Functional Enrichment of GCSC-Related Genes

To further investigate the principal biological functions associated with the GCSC-related risk model, GO enrichment analysis was performed for GCSC-associated genes, together with HALLMARK pathway enrichment analysis comparing the high- and low-risk groups. GO enrichment analysis revealed significant associations with key biological functions across three categories: biological process (BP), cellular component (CC), and molecular function (MF).

**Figure 6:**
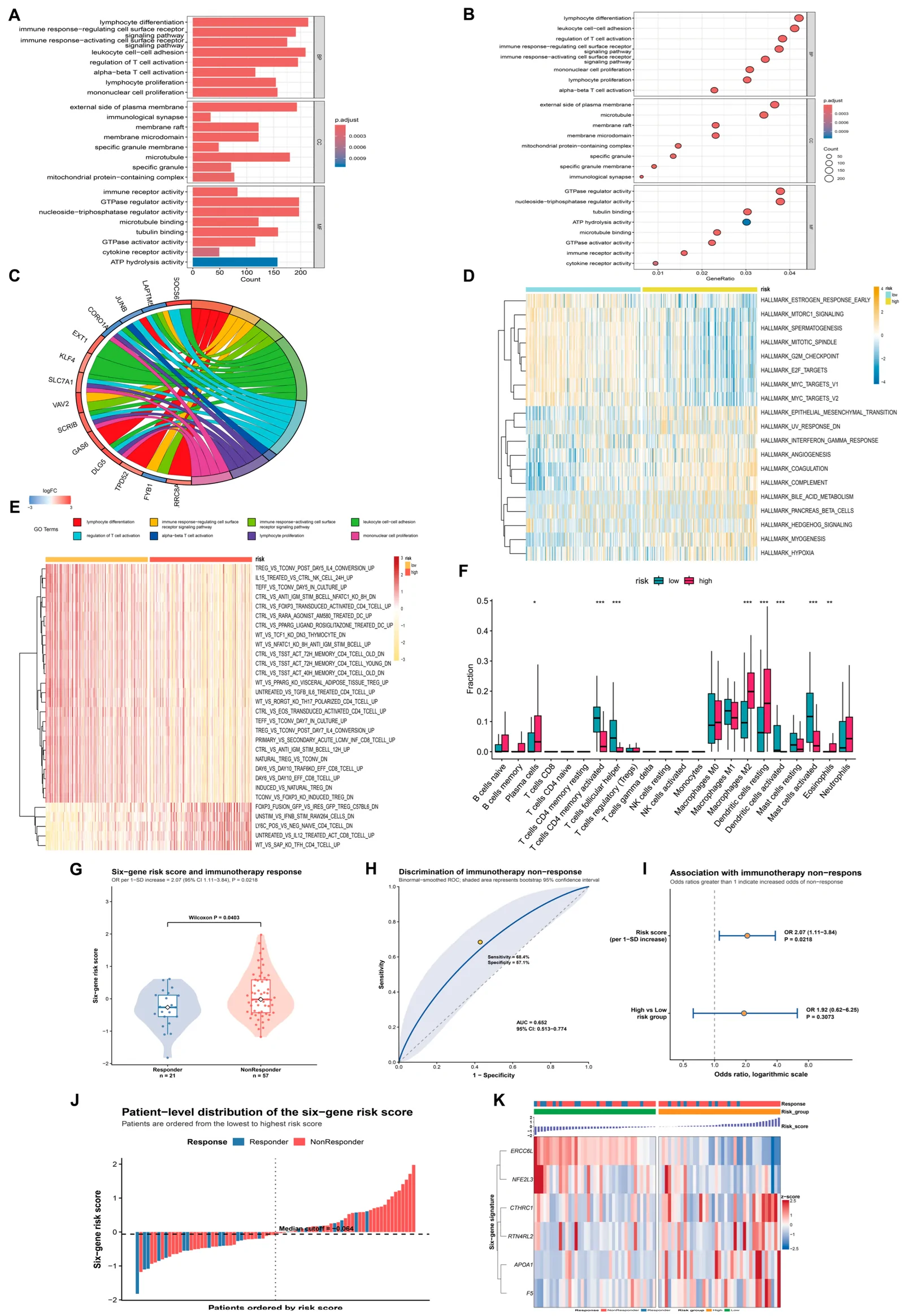
Functional enrichment and immune profiling of GCSC-related genes: (**A**,**B**) Bar plot and bubble plot of GO enrichment, listing significantly enriched GO terms associated with specific gene sets. (**C**) Chord diagram of GO enrichment, where colors represent pathways enriched by genes. (**D**) Heatmap of HALLMARK gene set enrichment analysis for high- and low-risk patient groups. (**E**) Heatmap of HALLMARK pathway activity in the high- and low-risk groups based on ssGSEA scores. Differential pathway activity was assessed using limma empirical Bayes-moderated t-tests with Benjamini-Hochberg FDR correction (adjusted *p* < 0.05 and |logFC| > 0.05). (**F**) Immune cell infiltration in the high- and low-risk groups. Differences in immune cell fractions were assessed using two-sided Wilcoxon rank-sum tests with multiple-testing correction. Statistical significance is indicated by * *p* < 0.05, ** *p* < 0.01, and *** *p* < 0.001. (**G**) Risk score distribution by immunotherapy response. (**H**) ROC curve for non-response prediction. (**I**) Univariable logistic regression forest plot. (**J**) Patient-level risk score waterfall plot. (**K**) Six-gene expression heatmap with response and risk group annotations.

Within the BP category, GCSC-associated genes were primarily enriched in lymphocyte differentiation, immune response regulating cell surface receptor signaling pathways, and leukocyte cell–cell adhesion (Figure 6A). These processes are essential for the establishment and regulation of the tumor immune microenvironment, suggesting that GCSC-associated genes may contribute to immune cell differentiation and immune-response-mediated biological activities.

Within the CC category, GCSC-associated genes were enriched in cellular structures such as the external side of the plasma membrane and microtubules, highlighting their potential involvement in intercellular interactions and structural organization. Within the MF category, significant enrichment was observed in GTPase regulator activity, nucleoside triphosphatase regulator activity, and immune receptor activity, indicating that GCSC-associated genes may play important roles in signal transduction and molecular interactions.

The GO bubble plot (Figure 6B) visualized the enrichment results using two key indicators: the gene ratio (GeneRatio) and adjusted *p* value (*p.adj*). These metrics facilitated identification of the most prominently enriched functional categories. For example, within the biological process category, lymphocyte differentiation exhibited a relatively high GeneRatio and a highly significant adjusted *p* value, indicating substantial enrichment of this biological process. The GO chord diagram further illustrates the specific functional pathways associated with individual genes. As shown in Figure 6C, most of these genes were involved in several immune-related biological processes, including lymphocyte differentiation and proliferation, immune cell surface receptor activation, regulation of T cell activation, and αβ T cell activation. These results provide a detailed view of the relationships between individual genes and their associated biological functions. The complete GO enrichment results and detailed gene–GO term relationships are provided in Supplementary Table S2 to facilitate further interpretation of these findings.

HALLMARK enrichment analysis (Figure 6D) revealed marked biological differences between the high- and low-risk groups. Several biological programs were more active in the high-risk group, particularly epithelial–mesenchymal transition (EMT), immune response pathways, inflammatory response pathways, and myogenesis. Enrichment of the EMT program suggests that tumors in high-risk patients may possess greater invasive and metastatic potential, consistent with the aggressive characteristics of gastric cancer. Enhanced immune and inflammatory signaling further indicates a more active but potentially dysregulated tumor immune microenvironment. Such an environment may contribute to immune evasion and tumor progression, although these associations require further experimental validation.

In contrast, the low-risk group was primarily enriched in E2F targets, the G2M checkpoint, MYC targets V1, and mTORC1 signaling. These pathways are closely associated with cell cycle progression, proliferation, and cellular growth, indicating that the low-risk group was characterized by a distinct proliferative program. For example, enrichment of the E2F target pathway reflects coordinated regulation of genes involved in cell cycle progression. However, pathway enrichment alone does not necessarily indicate stricter control of cell division and should therefore be interpreted cautiously.

Collectively, the GO and HALLMARK enrichment analyses revealed the potential biological functions underlying the GCSC-associated risk model in gastric cancer. The high-risk group exhibited biological features associated with invasion, metastasis, inflammation, and immune dysregulation, whereas the low-risk group was characterized predominantly by cell-cycle- and growth-related signaling programs. These findings provide a biological basis for understanding the potential roles of GCSC-associated genes in gastric cancer and may support the development of risk-adapted therapeutic strategies.

### 2.7. Immune Profiling of GCSC-Related Genes

Given that the cell–cell communication and HALLMARK enrichment analyses revealed strong associations between the GCSC-related risk model and immune cells and immune functions, single-sample gene set enrichment analysis (ssGSEA) and immune cell infiltration analyses were performed in the high- and low-risk groups to further characterize their immune microenvironment.

The ssGSEA results (Figure 6E) indicated that several immune-related processes were more prominent in the high-risk group. In particular, increased regulatory T cell (Treg) activity may suppress antitumor effector T cell responses and weaken immune-mediated tumor control. Macrophage-related alterations were also observed, with a shift toward tumor- promoting M2-like macrophage features, which may contribute to the establishment of a microenvironment favorable to tumor progression. Abnormal follicular helper T cell (Tfh) activity may further impair B cell-mediated antibody production and coordinated immune responses. In addition, reduced interferon-β (IFN-β) signaling may weaken antiviral and antitumor immune activity.

Collectively, these immune alterations suggest that the high-risk group is characterized by a more immunosuppressive tumor microenvironment. Such an environment may facilitate immune evasion, promote angiogenesis, and support tumor progression, thereby contributing to poorer treatment responses and survival outcomes.

Compared with the high-risk group, the low-risk group was characterized by enrichment of several immune-related processes, including effector T cell activation, enhanced natural killer (NK) cell activity, preservation of dendritic cell antigen presentation capacity, balanced regulation by regulatory T cells (Tregs), and maintenance of immunological memory. Activated effector T cells can directly eliminate tumor cells, whereas enhanced NK cell activity may improve the clearance of malignant cells. Dendritic cells (DCs) continuously present tumor-associated antigens and thereby support the initiation of T cell-mediated immune responses. Appropriate Treg activity may help maintain immune homeostasis without inducing excessive immunosuppression, while memory immune responses may provide sustained antitumor protection. Collectively, these coordinated immune processes suggest that the tumor microenvironment of low-risk patients may retain a relatively balanced immune surveillance system. Such a system may maintain antitumor immune pressure while limiting both excessive immunosuppression and uncontrolled inflammation, thereby restraining tumor progression.

Immune cell infiltration analysis (Figure 6F) further revealed marked differences in immune cell composition between the high- and low-risk groups. The high-risk group showed greater infiltration of resting dendritic cells, plasma cells, and M2-like macrophages. Resting dendritic cells may exhibit limited antigen-presenting capacity and therefore be less effective at activating antitumor T cell responses. Plasma cells may exert context-dependent effects within the tumor microenvironment and potentially contribute to immune dysregulation. M2-like macrophages are generally associated with immunosuppression, tissue remodeling, angiogenesis, and tumor progression.

In contrast, tumor tissues from low-risk patients exhibited higher infiltration of activated memory CD4+ T cells, follicular helper T cells (Tfh cells), and activated dendritic cells, all of which may support antitumor immunity. Activated memory CD4+ T cells can promote durable immune responses, while Tfh cells facilitate B cell activation and coordinated humoral immunity. Activated dendritic cells can more efficiently present tumor antigens and initiate antitumor T cell responses. These findings indicate that the risk model may not only stratify patients according to survival outcomes but also reflect differences in the composition and functional state of the tumor immune microenvironment.

To investigate the association between the six-gene risk score and immunotherapy response, the model was evaluated in the PRJEB25780 gastric cancer immunotherapy cohort, which included 78 patients comprising 21 responders and 57 non-responders.

The six-gene risk score was significantly higher in non-responders than in responders (Figure 6G). The Wilcoxon rank sum test confirmed a significant difference in score distribution between the two response groups (*p* = 0.0403), indicating that higher risk scores were associated with a greater likelihood of immunotherapy non-response.

Receiver operating characteristic analysis was subsequently performed to assess the ability of the six-gene score to distinguish non-responders from responders (Figure 6H). The area under the curve was 0.652, with a bootstrap 95% confidence interval of 0.515–0.778. These results indicate that the six-gene score provided modest discrimination between responders and non-responders.

Univariable logistic regression analysis was then conducted with immunotherapy non-response as the outcome (Figure 6I). Each one standard deviation increase in the risk score was associated with a 2.07-fold increase in the odds of non-response (OR = 2.07, 95% CI: 1.11–3.84, *p* = 0.0218). When patients were dichotomized into high- and low-risk groups according to the cohort median, the high-risk group had 1.92-fold higher odds of non-response than the low-risk group (OR = 1.92, 95% CI: 0.62–6.25, *p* = 0.3073). Although the dichotomized comparison did not reach statistical significance, the direction of the association was consistent with that observed in the continuous score analysis.

The patient-level waterfall plot showed the distribution of clinical responses across the ordered risk scores (Figure 6J). Non-responders became progressively enriched as the risk score increased, particularly among patients with the highest scores. This pattern further supported the association between an elevated six-gene risk score and immunotherapy non-response.

Finally, the six-gene expression heatmap illustrated the expression patterns of the model genes across individual patients (Figure 6K). The negatively weighted gene ERCC6L generally showed lower expression toward the high-risk region, whereas positively weighted genes such as CTHRC1 and RTN4RL2 tended to exhibit higher expression in high-risk patients. Non-responders were also more frequently observed in the high-risk region. These findings demonstrated directional consistency among the expression patterns of the six model genes, the composite risk score, and clinical immunotherapy response.

We next evaluated the six-gene risk score in the GSE91061 melanoma anti-PD-1 cohort, which included 49 pretreatment samples. Non-responders tended to have higher risk scores than responders (*p* = 0.115; Figure S3A). The score showed modest discrimination, with an AUC of 0.664 (95% CI: 0.431–0.887; Figure S3B). Logistic regression also showed directionally higher odds of non-response with increasing risk score and in the high-risk group (Figure S3C). The waterfall plot and gene expression heatmap further showed that non-responders were more frequently distributed in the high-risk region, with expression patterns broadly consistent with the model coefficients (Figure S3D,E).

In summary, ssGSEA and immune cell infiltration analyses demonstrated that the gene-based risk model was closely associated with the immune microenvironment of gastric cancer. The high-risk group was characterized by enrichment of immunosuppressive cell populations and functions, including regulatory T cells, M2 macrophages, and resting dendritic cells. In contrast, the low-risk group exhibited a relatively more active antitumor immune profile, with increased infiltration of activated memory CD4^+^ T cells, follicular helper T cells, and activated dendritic cells, indicating distinct immune states between the two risk groups. Further validation in immunotherapy cohorts showed that gastric cancer non-responders had significantly higher risk scores and that increasing risk scores were significantly associated with immunotherapy non-response. A directionally consistent trend was also observed in the melanoma cohort, although statistical significance was not reached because of the limited sample size. These findings suggest that the gene-based risk model not only reflects differences in the immune microenvironment of gastric cancer but may also have potential value for identifying patients who are unlikely to respond to immunotherapy. Nevertheless, its cross-cancer applicability requires further validation in larger independent cohorts.

### 2.8. Drug Sensitivity Analysis

**Figure 7:**
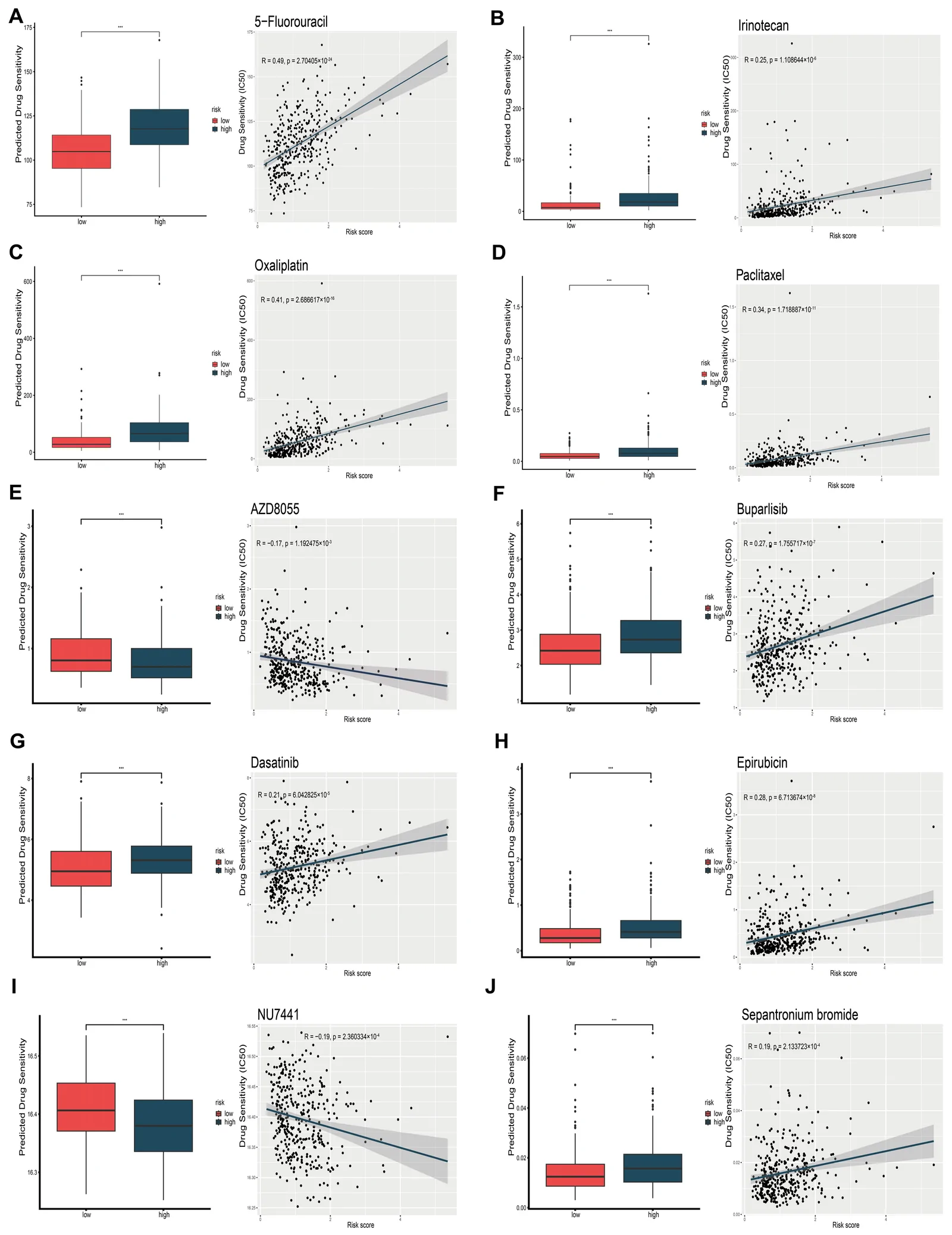
Drug sensitivity analysis: (**A**–**D**) Box plots and scatter plots showing predicted drug sensitivity and its correlation with risk scores for four clinically relevant chemotherapeutic agents: 5-fluorouracil, irinotecan, oxaliplatin, and paclitaxel. (**E**–**J**) Box plots and scatter plots showing predicted drug sensitivity and its correlation with risk scores for AZD8055, buparlisib, dasatinib, epirubicin, NU7441, and sepantronium bromide. Statistical significance is indicated by * *p* < 0.05, ** *p* < 0.01, and *** *p* < 0.001.

To further evaluate the potential of the risk model for predicting drug resistance in patients with gastric cancer, drug sensitivity analysis was performed in the high- and low-risk groups. Using the oncoPredict package, 18 drugs significantly associated with the risk model were identified. The complete drug sensitivity results for all 18 drugs are presented in Supplementary Figure S4. Representative drugs are shown in Figure 7A–J, revealing significant differences in predicted drug response between the two risk groups.

Given the clinical relevance of commonly used chemotherapeutic agents in gastric cancer treatment, we further evaluated the predicted sensitivity to 5-fluorouracil (5-FU), irinotecan, oxaliplatin, and paclitaxel. As shown in Figure 7A–D, patients in the high-risk group exhibited significantly higher predicted IC50 values for all four agents compared with those in the low-risk group. Furthermore, the risk score was positively correlated with the predicted IC50 values of 5-FU (R = 0.49, *p* < 0.001; Figure 7A), irinotecan (R = 0.25, *p* < 0.001; Figure 7B), oxaliplatin (R = 0.41, *p* < 0.001; Figure 7C), and paclitaxel (R = 0.34, *p* < 0.001; Figure 7D). These findings indicate that higher risk scores were associated with reduced predicted sensitivity to these clinically relevant chemotherapeutic agents.

Among the other evaluated agents, high-risk patients had higher predicted half-maximal inhibitory concentration values for several agents, including the phosphoinositide 3 kinase inhibitor buparlisib, the multitarget tyrosine kinase inhibitor dasatinib, epirubicin, and the survivin suppressant sepantronium bromide. Because a higher predicted IC50 indicates lower drug sensitivity, these findings suggest that high-risk patients may be less sensitive and potentially more resistant to these agents.

The PI3K signaling pathway plays an important role in cell growth, proliferation, survival, and metabolism and is frequently aberrantly activated in cancer. Buparlisib inhibits PI3K-mediated downstream signaling and thereby suppresses tumor cell proliferation and survival. As shown in Figure 7F, the risk score was positively correlated with the predicted IC50 of buparlisib (R = 0.27, *p* < 0.001), indicating that patients with higher risk scores were predicted to be less sensitive to buparlisib.

Epirubicin is an anthracycline that exerts antitumor effects by intercalating into DNA, inhibiting DNA and RNA synthesis, and generating free radicals that damage tumor cells. The predicted IC50 of epirubicin was also positively correlated with the risk score (Figure 7H, R = 0.28, *p* < 0.001), suggesting reduced epirubicin sensitivity in high-risk patients. Similar positive correlations were observed between the risk score and the predicted IC50 values of dasatinib (Figure 7G, R = 0.21, *p* < 0.001) and sepantronium bromide (Figure 7J, R = 0.19, *p* < 0.001). Collectively, these results suggest that increasing risk scores are associated with reduced predicted sensitivity to these drugs.

The analysis further identified several agents for which predicted drug sensitivity increased with the risk score. For the mTOR inhibitor AZD8055, the risk score was negatively correlated with the predicted IC50 (R = −0.17, *p* = 0.0012; Figure 7E). Because a lower IC50 indicates greater drug sensitivity, this result suggests that patients with higher risk scores may be more sensitive to AZD8055.

NU7441 primarily targets DNA-dependent protein kinase and interferes with the repair of DNA double-strand breaks. Its predicted IC50 was also negatively correlated with the risk score (R = −0.19, *p* < 0.001; Figure 7I), indicating that high-risk patients may exhibit greater predicted sensitivity to NU7441.

Collectively, the drug sensitivity analysis revealed distinct predicted response patterns between the high- and low-risk groups. High-risk patients were predicted to be less sensitive to several clinically used chemotherapeutic agents, including 5-FU, irinotecan, oxaliplatin, and paclitaxel, as well as whose IC50 values increased with the risk score, such as buparlisib, dasatinib, epirubicin, and sepantronium bromide. In contrast, they were predicted to be more sensitive to agents whose IC50 values decreased with the risk score, including AZD8055 and NU7441.

These findings suggest an association between the GCSC-related prognostic signature and predicted chemotherapy resistance, although they do not directly establish that GCSC abundance determines drug response. The risk model may help identify risk-group-specific drug sensitivity patterns and generate candidate therapeutic hypotheses for patients with gastric cancer. However, the results were derived from computational predictions and should not be used directly to guide drug selection or dose adjustment. Further validation in gastric cancer cell lines, patient-derived models, and prospective clinical cohorts is required before potential clinical application.

## 3. Discussion

GC remains a highly heterogeneous malignancy with substantial differences in tumor progression, treatment response, and patient survival. Previous single-cell transcriptomic studies have demonstrated extensive cellular and transcriptional heterogeneity in gastric cancer, providing a rationale for investigating specific malignant cell states rather than relying exclusively on bulk tumor expression profiles. Increasing evidence indicates that cancer stem cells represent a biologically distinct tumor cell population involved in tumor initiation, metastasis, recurrence, and therapeutic resistance [20,21]. However, conventional bulk transcriptomic approaches cannot accurately resolve the cellular origin and functional heterogeneity of stem-like tumor populations. In this study, we established a single-cell-informed GCSC-related prognostic framework by integrating scRNA-seq analysis with bulk transcriptomic cohorts. Through single-cell characterization of GCSC populations, functional investigation of their interactions with the tumor microenvironment, and translation of GCSC-associated molecular features into a patient-level risk model, we demonstrated that GCSC-related characteristics represent clinically meaningful determinants of gastric cancer progression and therapeutic response.

Our single-cell analysis identified a distinct GCSC population characterized by canonical stemness-associated markers, including CD44, CD24, KRT18, KRT8, and ALDH1A1. Importantly, the proportion of GCSCs varied substantially among individual gastric cancer samples, highlighting the intrinsic heterogeneity of stem-like tumor states across patients. Compared with conventional gastric cancer cells, GCSCs exhibited distinct transcriptional profiles, with higher expression of epithelial structural and stemness-associated genes. These findings suggest that GCSCs may not simply represent highly proliferative tumor cells but rather constitute a specialized cellular state characterized by self-renewal capacity and adaptive survival mechanisms. This interpretation is consistent with previous observations that chemotherapy-resistant gastric cancer cells can exhibit relatively reduced proliferative activity while retaining stem-like properties [22]. Previous studies have demonstrated that gastric cancer stem-like cells contribute to tumor initiation, EMT, invasion, metastasis, and resistance to conventional therapies. Consistent with these observations, our GCSC-related genes were enriched in biological processes associated with immune regulation, cellular interaction, and signaling regulation, suggesting that GCSC characteristics are closely linked to both intrinsic tumor properties and interactions with the surrounding microenvironment.

Unlike isolated tumor populations, GCSCs exist within a complex ecosystem composed of immune cells, stromal cells, and other malignant populations. Our cell–cell communication analysis revealed extensive interactions between GCSCs and multiple cellular components, particularly immune cell populations such as macrophages, neutrophils, T cells, and B cells. Previous studies have described bidirectional interactions between GCSCs and immune cells, suggesting that immune-associated signaling may contribute to stemness maintenance, immune evasion, and tumor progression [23]. The involvement of pathways including EGF, HGF, TENASCIN, COLLAGEN, LAMININ, and CDH suggests that GCSCs may actively participate in extracellular signaling networks that regulate stemness maintenance, cell adhesion, and tumor microenvironment remodeling.

In addition to intercellular communication, metabolic adaptation represents another important feature of CSC biology. Our metabolic analysis demonstrated that GCSCs displayed higher enrichment scores across multiple metabolic pathways, including glycolysis, oxidative phosphorylation, fatty acid degradation, and the tricarboxylic acid cycle. These metabolic characteristics may support the adaptability of GCSCs to heterogeneous and nutrient-limited tumor microenvironments. The concurrent enrichment of glycolysis- and oxidative phosphorylation-related pathways suggests that GCSCs may exhibit distinct metabolic adaptations rather than relying exclusively on a single energy-producing pathway. Previous experimental studies have demonstrated that the MYC/PGC-1α balance regulates the metabolic phenotype and oxidative phosphorylation dependence of pancreatic cancer stem cells [24]. Similarly, Valle et al. reported that enhanced oxidative phosphorylation was associated with increased stemness-associated properties, reversible quiescence, and immune evasion in pancreatic cancer stem cells [25]. These findings highlight mitochondrial metabolism as a potential therapeutic vulnerability in certain CSC populations. Further supporting the therapeutic relevance of mitochondrial metabolism, Melones-Herrero et al. demonstrated that novel platinum iodido compounds disrupted mitochondrial metabolism and reduced oxidative phosphorylation activity in gastrointestinal cancer cells, including gastric cancer cell lines, accompanied by oxidative stress and cellular senescence [26]. Together, these findings provide a biological context for the metabolic characteristics observed in GCSCs and highlight the potential relevance of mitochondrial metabolism as a therapeutic target. Nevertheless, further functional studies are required to determine whether GCSCs exhibit a direct dependence on oxidative phosphorylation and whether targeting these metabolic pathways can effectively impair their survival.

A major strength of this study is that the prognostic model was derived from GCSC-associated features identified at single-cell resolution rather than from bulk tumor expression profiles alone. Unlike conventional prognostic signatures that primarily capture averaged molecular changes across heterogeneous tumor tissues, our approach links a clinically applicable risk score with a specific cellular state associated with tumor stemness.

The six genes incorporated into the model, including NFE2L3, APOA1, CTHRC1, ERCC6L, RTN4RL2, and F5, may reflect multiple biological aspects of GCSC-associated tumor behavior. Among these genes, CTHRC1 has been reported to participate in extracellular matrix remodeling, tumor invasion, and malignant progression, Experimental studies in gastric cancer have demonstrated that CTHRC1 promotes migration and invasion, including through HIF-1α/CXCR4-associated signaling [27]. APOA1 is involved in lipid metabolism and metabolic regulation, which may be relevant to the metabolic adaptation observed in GCSCs. Previous research has also identified APOA1 as a potential diagnostic and prognostic biomarker in gastric cancer [28]. ERCC6L has been associated with cell proliferation and genome maintenance, processes that may contribute to tumor growth and treatment resistance. In particular, ERCC6L has been shown to promote gastric cancer growth and metastasis through NF-κB-associated signaling [29]. NFE2L3 has similarly been implicated in gastric cancer proliferation, epithelial–mesenchymal transition, and metastatic behavior [30]. Together, these genes represent different aspects of GCSC biology, including extracellular remodeling, metabolic adaptation, proliferation regulation, and tumor aggressiveness. The risk model showed robust prognostic performance across multiple independent cohorts and remained an independent prognostic factor after adjustment for conventional clinicopathological variables. These findings indicate that GCSC-related molecular features capture clinically relevant tumor characteristics beyond traditional staging systems.

Beyond survival prediction, our findings suggest that the GCSC-related risk score reflects differences in tumor immune states. High-risk patients exhibited enrichment of immunosuppressive immune characteristics, including increased M2-like macrophage signatures, regulatory T cell activity, and resting dendritic cell infiltration. In contrast, low-risk patients displayed enhanced antitumor immune features, including activated memory CD4+ T cells, activated dendritic cells, and immune-surveillance-related functions. These results are consistent with previous evidence that CSC populations can actively reshape immune microenvironments through cytokine secretion, metabolic regulation, and cell–cell interactions. An immunosuppressive niche may protect CSCs from immune elimination and facilitate tumor progression. Therefore, the association between the GCSC-related risk score and immune remodeling suggests that the model reflects not only tumor intrinsic stemness features but also the ecological interactions between tumor cells and immune components.

The relationship between GCSC characteristics and therapeutic resistance is another important implication of this study. Our immunotherapy analyses demonstrated that higher risk scores were associated with increased likelihood of immunotherapy non-response in the gastric cancer cohort, with a similar directional trend observed in the melanoma anti-PD-1 cohort. Although further validation is required, these findings suggest that GCSC-related features may influence immune checkpoint therapy responsiveness. Furthermore, drug sensitivity analysis indicated that high-risk patients showed reduced predicted sensitivity to several clinically relevant chemotherapy agents, including 5-fluorouracil, oxaliplatin, irinotecan, and paclitaxel, as reflected by increased predicted IC50 values. This observation is biologically plausible because CSC populations are frequently associated with resistance to cytotoxic therapies through enhanced DNA repair capacity, metabolic adaptation, and survival signaling. Therefore, the GCSC-related risk score may represent a potential indicator of a treatment-resistant phenotype and may help identify patients who require alternative therapeutic strategies.

Collectively, this study provides a comprehensive framework connecting single-cell-defined GCSC characteristics with patient-level clinical outcomes. Rather than simply identifying prognostic genes, our approach reveals a biological continuum from GCSC cellular states to molecular signatures, immune remodeling, and therapeutic response patterns. This framework may provide new insights into the role of GCSCs in gastric cancer progression and support future development of personalized treatment strategies.

Several limitations should be acknowledged. First, although multiple independent cohorts were used for validation, all analyses were based on retrospective public datasets, and prospective multicenter studies are required to confirm the clinical utility of the model. Second, the biological functions of the six genes and their direct roles in maintaining GCSC properties require experimental validation using cellular and animal models. Third, drug sensitivity and immunotherapy response analyses were based on computational predictions and limited clinical cohorts; therefore, these findings should be interpreted cautiously and require further experimental and clinical confirmation. Furthermore, the present study primarily focused on gene-level transcriptomic features and did not comprehensively characterize transcript isoforms, alternative splicing, or RNA modifications. Recent advances in single-cell sequencing technologies, nanopore-based sequencing approaches, and direct RNA sequencing of native RNA molecules offer new opportunities to investigate these additional layers of transcriptomic regulation. Future studies integrating long-read single-cell transcriptomics, native RNA sequencing, and functional validation may provide a more comprehensive understanding of transcript-level regulatory complexity and its potential contribution to GCSC heterogeneity, tumor progression, and therapeutic response [31,32].

## 4. Materials and Methods

### 4.1. Data Acquisition and Preprocessing

This study integrated single-cell transcriptomic data, bulk transcriptomic data, clinical follow up information, and immunotherapy cohorts. All datasets were obtained from publicly available databases.

The scRNA-seq dataset GSE163558 was downloaded from the GEO database and included 33,989 cells from 10 gastric cancer samples. Following doublet identification and removal, 31,820 singlet cells were retained for downstream single-cell analyses. This dataset was used for cell subpopulation identification, cell–cell communication analysis, and metabolic characterization.

Transcriptomic and clinical data for gastric cancer were obtained from TCGA-STAD cohort. Fragments per kilobase of transcript per million mapped read values were converted to transcripts per million and subsequently transformed using log2(TPM+1). Of the 448 initially available cases, three were excluded because of incomplete clinical or survival information. Consequently, 445 patients, including 173 deaths (OS events) and 272 censored observations, were included in the final training cohort for prognostic model construction. Patients with complete overall survival and essential clinical information were retained for prognostic model construction.

GSE84437 and GSE62254, comprising 483 and 300 gastric cancer samples, respectively, were used as independent prognostic validation cohorts. Gene identifiers were harmonized across datasets, and model gene expression values were standardized before validation.

For external validation, the GSE84437 and GSE62254 expression matrices were processed independently. Log transformation was applied where necessary, followed by gene-wise Z-score standardization across samples within each validation cohort. The same six prognostic genes and their corresponding Cox regression coefficients derived from the TCGA-STAD training cohort were applied to both external validation cohorts without coefficient re-estimation. Risk scores were calculated using the established six-gene signature. Patients were subsequently classified into high- and low-risk groups according to the median risk score calculated separately within each cohort, rather than using a fixed cutoff derived from the training cohort. KM survival analysis and time-dependent ROC curves were used to evaluate the prognostic performance of the model in the external validation cohorts.

The PRJEB25780 gastric cancer immunotherapy cohort and the GSE91061 melanoma anti-PD-1 cohort were used to evaluate the association between the six-gene risk score and immunotherapy response. PRJEB25780 included 78 patients with available response information. For GSE91061, only pretreatment samples with complete response information were included, resulting in 49 patients.

### 4.2. Single-Cell RNA Sequencing Data Processing and Cell Annotation

The GSE163558 expression matrix was analyzed using the Seurat package (version 5.1.0) [33]. Genes detected in fewer than three cells and cells with fewer than 300 detected genes were excluded during Seurat object construction. Additional quality control criteria included 300–5000 detected genes per cell, mitochondrial gene expression below 10%, hemoglobin-related gene expression below 3%, and total unique molecular identifier counts above 1000 and below the 97th percentile of the corresponding sample.

Potential doublets were identified using the scDblFinder package (version 1.18.0), with sample-specific identification performed using the original sample identifiers (orig.ident). Default parameters were used unless otherwise specified. Of the 33,989 cells passing initial quality control, 2169 predicted doublets (6.38%) were excluded, resulting in 31,820 singlet cells for subsequent analyses.

Following quality control and doublet removal, the samples were merged and log normalized using the NormalizeData function. A total of 3000 highly variable genes were identified using the “vst” method, followed by data scaling and principal component analysis using 30 principal components. Batch effects among samples were corrected using Harmony (version 1.2.0) [34] with orig.ident as the batch variable. The first 11 Harmony dimensions were used for nearest neighbor graph construction and unsupervised clustering at a resolution of 0.5. Uniform manifold approximation and projection and t-distributed stochastic neighbor embedding were used for visualization.

Cluster-specific marker genes were identified using the FindAllMarkers function with the Wilcoxon rank sum test, with only.pos = TRUE and logfc.threshold = 0.25. Cell types were annotated according to canonical marker genes and cluster-specific expression profiles.

### 4.3. Cell–Cell Communication Inference Using CellChat

Cell–cell communication was analyzed using the CellChat package (version 1.5.0) [35]. The normalized RNA expression matrix and cell-type annotations were used as input, and CellChatDB.human was used as the reference ligand receptor database.

The CellChat object was constructed from the normalized expression data in the Seurat RNA assay, with annotated cell types specified as the grouping variable. Signaling-related genes and overexpressed ligand–receptor interactions were identified using the identifyOverExpressedGenes and identifyOverExpressedInteractions functions. Protein–protein interaction network projection was performed using PPI.human. Communication probabilities were calculated using computeCommunProb with raw.use = TRUE and the default triMean method, based on the normalized, non-projected expression data. The default settings of 100 bootstrap iterations and population.size = FALSE were retained. Cell groups containing fewer than 10 cells were excluded from communication inference using filterCommunication (min.cells = 10). Parallel processing was configured with five workers.

Communication probabilities were calculated to construct ligand receptor and signaling-pathway networks among the annotated cell populations. Pathway-level communication probabilities were calculated using computeCommunProbPathway, followed by network aggregation using aggregateNet. Network centrality was evaluated using netAnalysis_computeCentrality. Analyses focused on the communication strength, major ligand receptor interactions, and signaling pathways between GCSCs and tumor, immune, and stromal cells.

Incoming and outgoing communication patterns were identified using non-negative matrix factorization. The number of communication patterns was explored using selectK, and five patterns (k = 5) were specified for both outgoing and incoming signaling analyses. The number of communication patterns was selected using cophenetic and silhouette coefficients and visualized using heatmaps, network plots, and river plots.

### 4.4. Single-Cell Metabolic Pathway Analysis

Metabolic pathway activity was evaluated using the scMetabolism package (version 0.2.2) and Kyoto Encyclopedia of Genes and Genomes metabolic gene sets [36,37]. AUCell was used to calculate pathway activity scores for individual cells [38].

Specifically, metabolic pathway scoring was performed using the sc.metabolism.Seurat function with metabolism.type = “KEGG”, method = “AUCell”, imputation = FALSE, and ncores = 4. Pathway activity scores were subsequently averaged across cells within each cluster and visualized using heatmaps. For heatmap visualization, pathway scores were Z-score standardized across cell clusters.

Metabolic activities were compared between GCSCs and non-GCSC tumor cells, with particular attention to oxidative phosphorylation, glycolysis, lipid metabolism, and amino acid metabolism. Differences between groups were evaluated using the Wilcoxon rank sum test, and *p* values were adjusted using the Benjamini–Hochberg method. Pathways with adjusted *p* < 0.05 were considered statistically significant.

### 4.5. Identification and Functional Analysis of GCSC-Related Genes

Differential expression analysis was performed between GCSCs and non-GCSC tumor epithelial cells using the Wilcoxon rank sum test. Genes with ∣log2FC∣ ≥ 1 and *P*_adj_ < 0.05 were considered GCSC-related differentially expressed genes.

Gene identifiers were converted using the org.Hs.eg.db package (version 3.18.0) [39]. Gene Ontology and Kyoto Encyclopedia of Genes and Genomes enrichment analyses were performed using the clusterProfiler (version 4.8.3) package, and significantly enriched terms were defined by adjusted *p* < 0.05. Single-sample gene set enrichment analysis was additionally performed using HALLMARK gene sets from the Molecular Signatures Database [40,41]. Pathway activity scores were compared between the high- and low-risk groups to investigate differences in epithelial–mesenchymal transition, proliferation, inflammation, and metabolic processes.

### 4.6. Construction and Validation of the Six-Gene Prognostic Risk Model

The TCGA-STAD cohort was used as the training cohort, whereas GSE62254 and GSE84437 were used as independent validation cohorts [42]. Univariable Cox proportional hazards regression was first performed to identify GCSC-related genes associated with overall survival. Genes with *p* < 0.001 were retained as candidate prognostic genes for subsequent modeling.

Candidate genes were subsequently entered into least absolute shrinkage and selection operator Cox regression [43]. LASSO-Cox regression was implemented using the glmnet package (version 4.1.3) with family = “cox” and alpha = 1. Ten-fold cross-validation (nfolds = 10) was performed using partial likelihood deviance (type.measure = “deviance”) to determine the optimal regularization parameter. The minimum cross-validation error criterion (lambda.min) was used for gene selection, and nonzero coefficients were extracted from the fitted glmnet model at the selected lambda value. Predictor standardization within glmnet followed the package default setting. Multivariable Cox regression was then used to identify independent prognostic genes and establish the final six-gene model.

The risk score was calculated as follows:

$$\mathrm{Risk}\;\mathrm{Score}=\sum_{K=1}^{N}\left(\mathrm{Expk}*\mathrm{Coef}\right)$$

where ***Coef*** represents the Cox regression coefficient of the k-th gene and ***Expk*** represents its corresponding preprocessed expression value used for risk-score calculation.

Patients were classified into high- and low-risk groups according to the median risk score. Overall survival was compared using KM curves and the log rank test. Time-dependent receiver operating characteristic curves were generated using the timeROC package to evaluate 1-, 3-, and 5-year predictive performance [44].

Risk scores in the validation cohorts were calculated using the same model coefficients. KM and time-dependent receiver operating characteristic analyses were used to assess model performance in the independent cohorts.

Univariable and multivariable Cox regression analyses were performed to determine whether the risk score was independent of available clinical variables. A nomogram was constructed based on the multivariable model [45], and calibration curves were used to evaluate predicted and observed survival probabilities at 1, 3, and 5 years.

### 4.7. Immune Microenvironment Analysis

Only TCGA-STAD tumor samples were included in the immune microenvironment analysis. ssGSEA was used to estimate immune cell and immune function scores.

The CIBERSORT (version 0.1.0) algorithm with the LM22 signature matrix was used to estimate the relative proportions of 22 immune cell populations [46]. Before CIBERSORT analysis, the expression matrix was processed using the voom function from the limma package (version 3.56.2). CIBERSORT was performed with 1000 permutations (perm = 1000), and quantile normalization was disabled (QN = FALSE). Samples were matched to the corresponding prognostic risk-score data using sample identifiers. Only samples with a CIBERSORT deconvolution *p*-value < 0.05 were retained for subsequent immune infiltration analyses. Other CIBERSORT parameters were maintained at their default settings. Differences in immune cell proportions and immune function scores between the high- and low-risk groups were assessed using the Wilcoxon rank sum test, followed by Benjamini–Hochberg correction.

Heatmaps, box plots, and correlation plots were used to visualize associations among risk scores, model-gene expression, immune cell infiltration, and immune-related functions.

### 4.8. Validation in Immunotherapy Cohorts

The six-gene risk model was evaluated in the PRJEB25780 gastric cancer immunotherapy cohort and the GSE91061 melanoma anti-PD-1 cohort. Only pretreatment patients with complete expression data for all six model genes and clearly defined treatment responses were included.

Patients with complete or partial responses were classified as responders, whereas those with stable or progressive disease were classified as non-responders [47]. Non-response was defined as the event of interest, with non-responders coded as 1 and responders coded as 0.

The expression levels of the six genes were standardized within each cohort, and risk scores were calculated using the fixed model coefficients. Risk score distributions between responders and non-responders were compared using the Wilcoxon rank sum test.

The risk score was further standardized to evaluate the association between each one standard deviation increase and immunotherapy non-response using univariable logistic regression. Odds ratios, 95% confidence intervals, and *p* values were reported.

Receiver operating characteristic curve analysis was used to assess the discriminatory ability of the risk score. Areas under the curve and their 95% confidence intervals were calculated using 2000 stratified bootstrap resamples. Patients were also divided into high- and low-risk groups according to the median risk score within each cohort, and Fisher’s exact test was used to evaluate differences in non-response.

PRJEB25780 served as the gastric cancer immunotherapy validation cohort, whereas GSE91061 was considered an exploratory cross-cancer validation cohort.

### 4.9. Computational Prediction of Drug Sensitivity

Drug sensitivity in TCGA-STAD patients was predicted using the oncoPredict package (version 1.2) [48]. Predicted half maximal inhibitory concentrations were compared between the high- and low-risk groups.

Spearman correlation analysis was performed to assess associations between the risk score and predicted drug response. An initial threshold of *p* < 0.001 was applied, followed by Benjamini–Hochberg correction for multiple comparisons.

Box plots were used to display differences between risk groups, and scatter plots were used to visualize correlations between risk scores and predicted drug sensitivity. Lower predicted half maximal inhibitory concentrations indicated potentially greater drug sensitivity.

### 4.10. Statistical Analysis

All statistical analyses were performed using R software (version 4.3.0). A random seed of 1234 was specified in the analysis scripts to improve computational reproducibility. The R scripts used for the bioinformatics analyses in this study are publicly available at https://github.com/huh492735-collab/GCSC-Prognostic-Model (accessed on: 28 October 2026). Unless otherwise specified, analytical parameters were maintained at the default settings of the corresponding software packages. All statistical tests were two-sided unless otherwise specified.

Continuous variables were summarized as the mean and standard deviation or median and interquartile range, as appropriate. Comparisons between two groups were performed using the Wilcoxon rank sum test. Categorical variables were compared using the chi square test or Fisher’s exact test.

Survival differences were evaluated using KM analysis and the log rank test. Prognostic associations were assessed using Cox proportional hazards regression and reported as hazard ratios with 95% confidence intervals. Associations with immunotherapy non-response were evaluated using logistic regression and reported as odds ratios with 95% confidence intervals.

Receiver operating characteristic curve analysis was used to evaluate model discrimination. For analyses involving multiple genes, pathways, immune cell populations, or drugs, Benjamini–Hochberg adjustment was applied where specified in the corresponding analysis. *p* values were adjusted using the Benjamini–Hochberg method. Unless otherwise indicated, *p* < 0.05 or Padj < 0.05 was considered statistically significant.

## 5. Conclusions

In this study, scRNA-seq data were used to identify GCSCs and to investigate their potential roles within the TME. A GCSC-associated prognostic risk model was subsequently constructed and its predictive performance was evaluated.

The results revealed extensive and complex cell–cell communication between GCSCs and immune cells in the TME. These interactions may contribute to the establishment, maintenance, and dynamic remodeling of the tumor microenvironment. Pathway enrichment analysis further showed that GCSCs exhibited relatively high activity in metabolic pathways such as oxidative phosphorylation and glycolysis.

GCSC-associated differentially expressed genes were then subjected to a stepwise screening procedure. Six key genes, NFE2L3, APOA1, CTHRC1, ERCC6L, RTN4RL2, and F5, were ultimately identified. Based on these genes, a prognostic risk model for gastric cancer was constructed and validated in multiple independent datasets. The results showed that the model could reproducibly stratify patients with gastric cancer into high- and low-risk groups.

Functional enrichment analysis indicated that GCSC-associated genes were predominantly involved in immune-related biological pathways, suggesting that they may participate in the regulation of immune cell development and the initiation of immune responses. Immune infiltration analysis further showed that the TME of high-risk patients contained higher estimated levels of M2-like macrophages and neutrophils. Increased infiltration of these cells may contribute to the formation of an immunosuppressive microenvironment, thereby reducing the ability of the immune system to eliminate tumor cells.

Drug sensitivity analysis generated potential therapeutic hypotheses for patients in different risk groups. The results suggested that high-risk patients may exhibit lower predicted sensitivity to several drug classes, including PI3K inhibitors and anthracycline antibiotics. These findings may help prioritize candidate treatments for subsequent experimental and clinical evaluation.

Overall, this study integrated multi omics data to characterize the molecular features of GCSCs, investigate their interactions with the TME, and explore their potential roles in gastric cancer progression. On this basis, a prognostic risk model derived from GCSC-associated genes was established. Further prospective validation and functional experiments are required to confirm these findings and determine their potential value for improving the precision of prognostic assessment and treatment in gastric cancer.

## Figures and Tables

**Table 1:** Prognostic gene in the context.

Gene	Coef
NFE2L3	−0.1768
APOA1	0.0826
CTHRC1	0.1600
ERCC6L	−0.5076
RTN4RL2	0.1791
F5	0.1301

The first and second columns represent the prognostic genes and their corresponding regression coefficients, respectively.

## Data Availability

The original contributions presented in this study are included in the article/Supplementary Material. Further inquiries can be directed to the corresponding author. All datasets analyzed in this study were obtained from previously published and publicly available resources. The scRNA-seq dataset GSE163558 was obtained from the GEO database and used for single-cell analyses. Bulk transcriptomic and clinical data from TCGA-STAD were used for prognostic model development. GSE62254 and GSE84437 were used as independent prognostic validation cohorts. The PRJEB25780 gastric cancer immunotherapy cohort and the GSE91061 melanoma anti-PD-1 cohort were used for immunotherapy response validation. GSE163558, GSE62254, GSE84437, and GSE91061 are publicly accessible through GEO; TCGA-STAD is available through the National Cancer Institute Genomic Data Commons; and PRJEB25780 is available through the European Nucleotide Archive. No new sequencing data were generated in this study.

## Supplementary Materials

The following supporting information can be downloaded at: https://www.mdpi.com/article/10.3390/ijms27219669/s1.

## Abbreviations

The following abbreviations are used in this manuscript:

GC	Gastric Cancer
GCSCs	Gastric Cancer Stem Cells
TME	Tumor Microenvironment
PCA	Principal Component Analysis
ROC	Receiver Operating Characteristic
EMT	Epithelial-mesenchymal transition
ssGSEA	single-sample Gene Set Enrichment Analysis
TCGA-STAD	The Cancer Genome Atlas Stomach Adenocarcinoma
