# Supplementary material for: Construction of a GCSCs-Related Prognostic Risk Model Based on Transcriptomic Data
Source: Int J Mol Sci. 2026 Oct 28;27(21):9669. doi: 10.3390/ijms27219669 (PMC13763221; doi:10.3390/ijms27219669)
Supplement: Supplementary file 1 [file ijms-27-09669-s001.zip › Supplementary Fig.pdf]

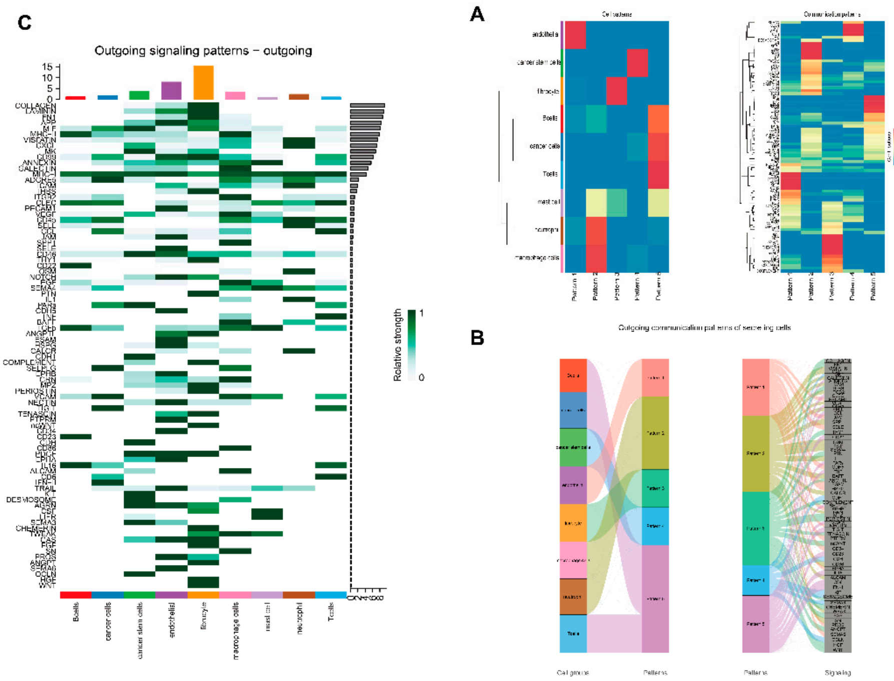
C
Outgoing signaling patterns − outgoing
Relative strength
Bcells
cancer cells
cancer stem cells
endothelial
fibrocyte
macrophage cells
mast cell
neutrophil
Tcells
A
B
Outgoing communication patterns of secreting cells
Cell groups
Patterns
Signaling

Figure S1. Cell–cell communication and metabolic functional analysis of GCSCs (A) Heatmaps showing outgoing signaling patterns among the nine cell types. (B) Sankey diagrams illustrating the outgoing signaling patterns among the nine cell types. (C) Clustering heatmaps showing outgoing signals across cell types.

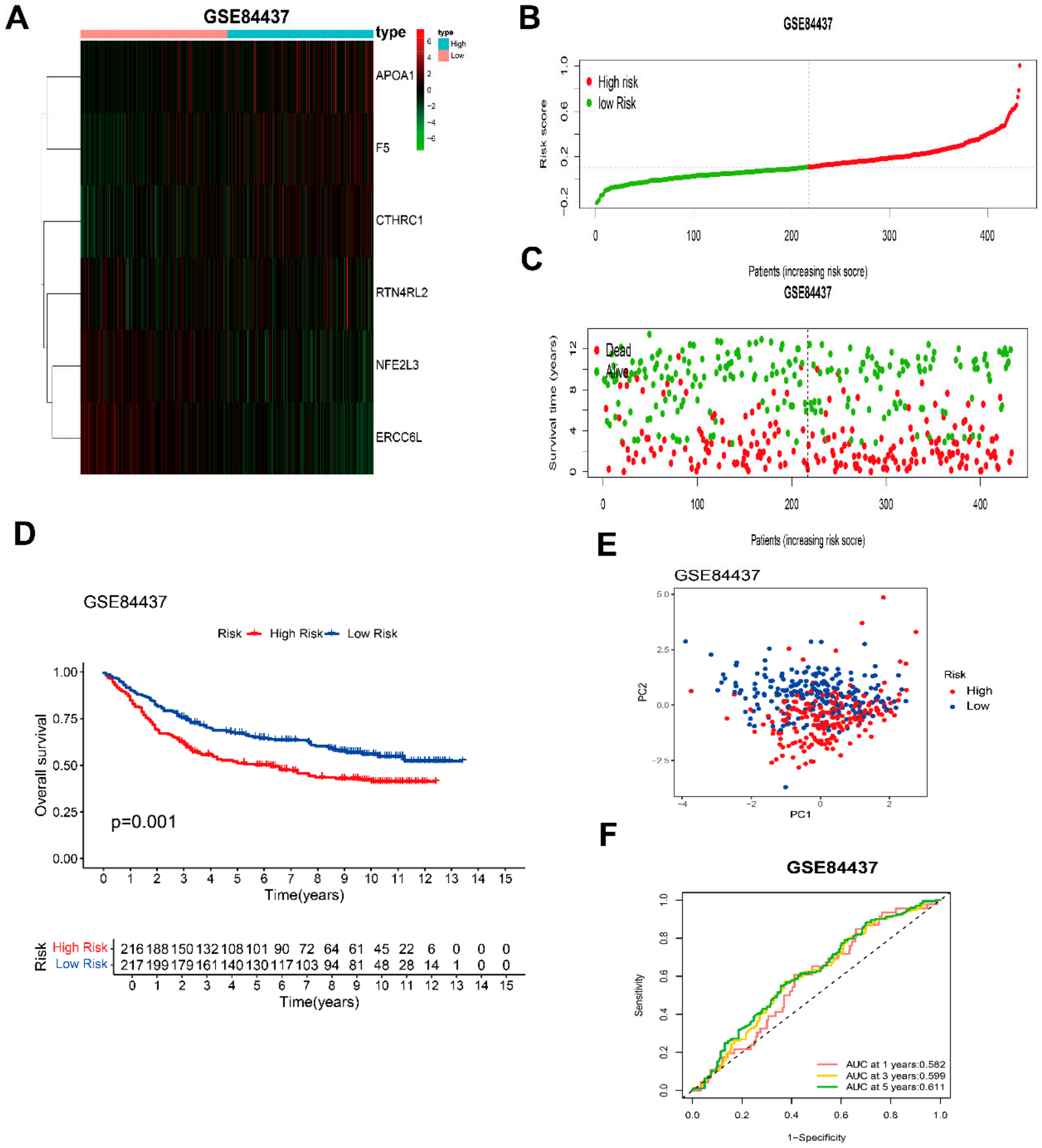

A
GSE84437
type
APOA1
F5
CTHRC1
RTN4RL2
NFE2L3
ERCC6L
High
Low
B
GSE84437
High risk
low Risk
Risk score
Patients (increasing risk socre)
C
GSE84437
Dead
Alive
Survival time (years)
Patients (increasing risk socre)
D
GSE84437
Risk
High Risk
Low Risk
Overall survival
p=0.001
Time(years)
High Risk 216 188 150 132 108 101 90 72 64 61 45 22 6 0 0 0
Low Risk 217 199 179 161 140 130 117 103 94 81 48 28 14 1 0 0
Time(years)
E
GSE84437
PC1
PC2
Risk
High
Low
F
GSE84437
Sensitivity
1−Specificity
AUC at 1 years:0.582
AUC at 3 years:0.599
AUC at 5 years:0.611

Figure S2. Validation of the GCSC Related Gene Prognostic Risk Model (A) Risk heatmap of GSE84437, where the top color bar distinguishes high and low risk patients. (B) Risk score distribution plot for GSE84437, with patients ordered by ascending risk scores. (C) Risk scatter plot correlating risk scores with survival outcomes in the GSE84437 cohort. (D) KM survival curves for the GSE84437 cohort. (E) Time dependent ROC curves for 1, 3, and 5 year survival prediction in the GSE84437 cohort. (F) PCA plots of high and low risk samples in the GSE84437 cohort.

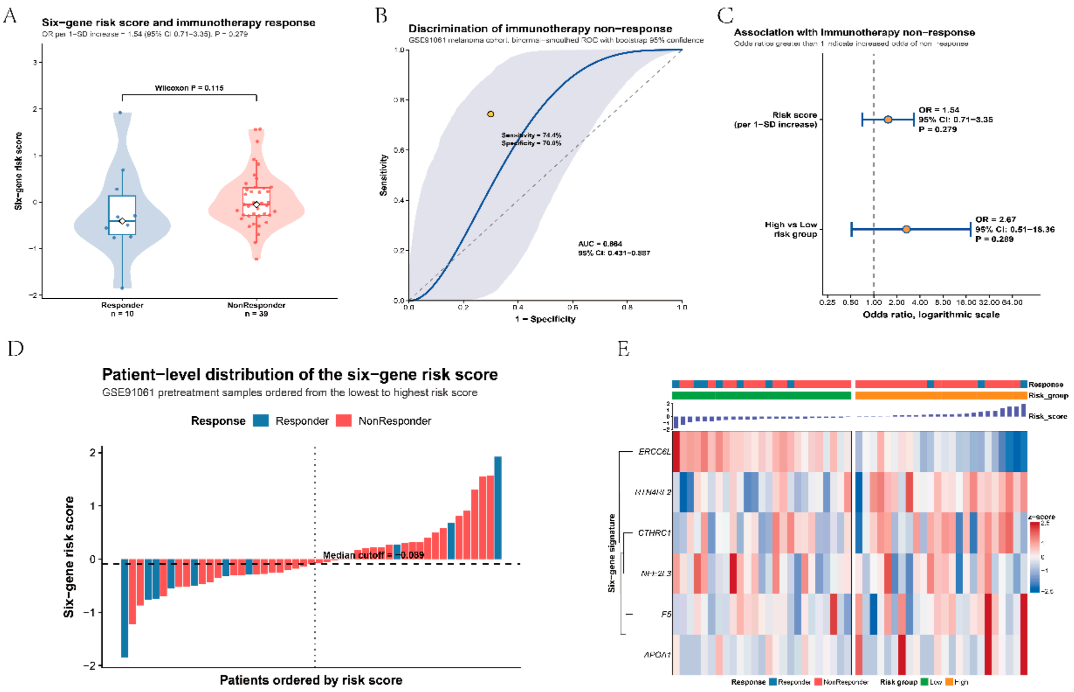
A
Six−gene risk score and immunotherapy response
OR per 1−SD increase = 1.54 (95% CI 0.71–3.35), P = 0.279
Wilcoxon P = 0.115
Six−gene risk score
Responder
n = 10
NonResponder
n = 39
B
Discrimination of immunotherapy non−response
GSE91061 melanoma cohort, binormal−smoothed ROC with bootstrap 95% confidence
Sensitivity = 74.4%
Specificity = 70.0%
AUC = 0.664
95% CI: 0.431–0.887
Sensitivity
1 − Specificity
C
Association with immunotherapy non−response
Odds ratios greater than 1 indicate increased odds of non−response
Risk score
(per 1−SD increase)
OR = 1.54
95% CI: 0.71−3.35
P = 0.279
High vs Low
risk group
OR = 2.67
95% CI: 0.51−18.36
P = 0.289
0.25 0.50 1.00 2.00 4.00 8.00 16.00 32.00 64.00
Odds ratio, logarithmic scale
D
Patient−level distribution of the six−gene risk score
GSE91061 pretreatment samples ordered from the lowest to highest risk score
Response Responder NonResponder
Six−gene risk score
Median cutoff = −0.089
Patients ordered by risk score
E
Response
Risk_group
Risk_score
Six−gene signature
ERCC6L
CTHRC1
NFE2L3
F5
APOA1
z−score
Response Responder NonResponder Risk group Low High

Figure S3. Immune Profiling of GCSC Related Genes (A) Risk score distribution between responders and non responders. (B) ROC curve for immunotherapy non response. (C) Univariable logisticregression forest plot. (D) Patient level risk score distribution. (E) Six gene expression heatmap with response and risk group annotations.

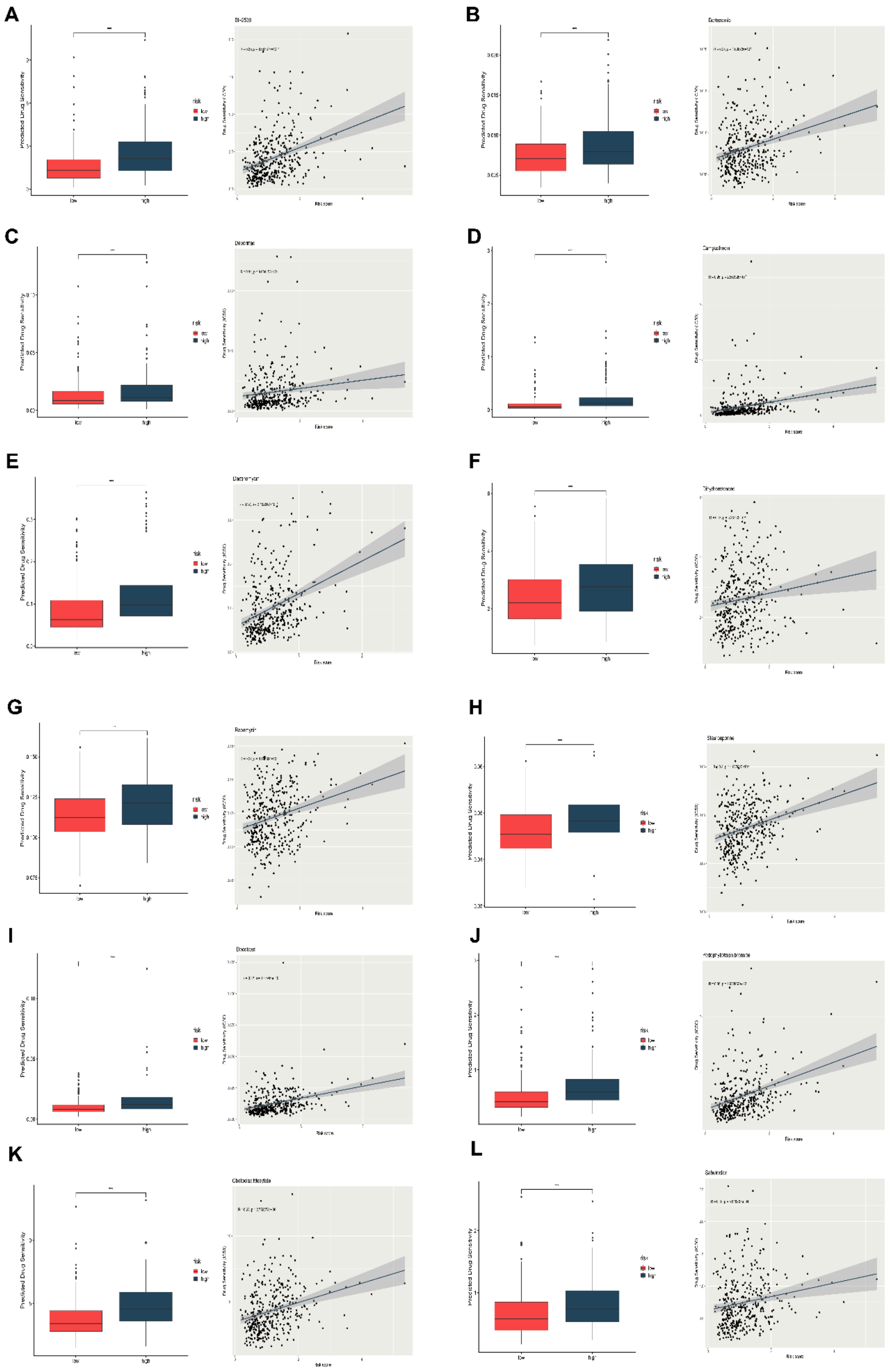
A
B
C
D
E
F
G
H
I
J
K
L
Predicted Drug Sensitivity
risk
low
high

Figure S4. Drug Sensitivity Analysis (A-L) Box plots and scatter plots showing predicted drug sensitivity and its correlation with risk scores for BI-2536, Bortezomib, Daporinad, Camptothecin, Dactinomycin, Dihydrorotenone, Rapamycin, Staurosporine, Docetaxel, Podophyllotoxin bromide, Obatoclax Mesylate, and Sabutoclax.
