# Supplementary material for: Construction of a GCSCs-Related Prognostic Risk Model Based on Transcriptomic Data
Source: Int J Mol Sci. 2026 Oct 28;27(21):9669. doi: 10.3390/ijms27219669 (PMC13763221; doi:10.3390/ijms27219669)
Supplement: Supplementary file 1 [file ijms-27-09669-s001.zip › Supplementary Table S1.pdf]

Supplementary Table S1:

Results of univariate Cox regression analysis .

Gene Symbol	HR	HR.95L	HR.95H	P-value
NFE2L3	0.751099848	0.621370932	0.907913379	0.003090763
POLQ	0.676732903	0.518478706	0.883290706	0.004064933
RAD54L	0.692295294	0.523808485	0.914977111	0.009754021
ANGPTL1	1.169781245	1.043511523	1.311330188	0.007128495
MFAP2	1.201336046	1.050825804	1.373403936	0.0072342
APOA1	1.094910935	1.027320013	1.166948897	0.005286579
MYB	0.789804795	0.677165084	0.921181007	0.002649568
STIL	0.73174466	0.590924582	0.906122818	0.004184362
BRIP1	0.55414784	0.384263995	0.799137657	0.001575741
TMOD1	1.250549499	1.075407622	1.454215144	0.003680408
CEP55	0.78605063	0.655564301	0.942509516	0.009342508
COL8A1	1.209921479	1.072472477	1.364986065	0.001953889
CTHRC1	1.204199378	1.066539577	1.359627129	0.002699513
DDIAS	0.687921735	0.527281013	0.897503043	0.005834563
ESCO2	0.673664923	0.521661866	0.869958987	0.002464009
CDCA2	0.757963459	0.622048174	0.923575746	0.005987479
PARPBP	0.676842374	0.505036607	0.907093848	0.008984013
KIF24	0.648079094	0.483777468	0.868181219	0.003643206
ERCC6L	0.594650773	0.441474507	0.800973864	0.000625506
RTN4RL2	1.244691219	1.054121836	1.469712681	0.009833882
F5	1.16017399	1.045172947	1.287828671	0.005278548
